# Long-term trends in mortality and AIDS-defining events after combination ART initiation among children and adolescents with perinatal HIV infection in 17 middle- and high-income countries in Europe and Thailand: A cohort study

**DOI:** 10.1371/journal.pmed.1002491

**Published:** 2018-01-30

**Authors:** The European Pregnancy and Paediatric HIV Cohort Collaboration (EPPICC) study group in EuroCoord, Ali Judd, Elizabeth Chappell, Anna Turkova, Sophie Le Coeur, Antoni Noguera-Julian, Tessa Goetghebuer, Katja Doerholt, Luisa Galli, Dasja Pajkrt, Laura Marques, Intira J. Collins, Diana M. Gibb, Maria Isabel González Tome, Marisa Navarro, Josiane Warszawski, Christoph Königs, Vana Spoulou, Filipa Prata, Elena Chiappini, Lars Naver, Carlo Giaquinto, Claire Thorne, Magdalena Marczynska, Liubov Okhonskaia, Klara Posfay-Barbe, Pradthana Ounchanum, Pornchai Techakunakorn, Galina Kiseleva, Ruslan Malyuta, Alla Volokha, Luminita Ene, Ruth Goodall

**Affiliations:** 1 MRC Clinical Trials Unit, University College London (UCL), London, United Kingdom; 2 Institut National d'Etude Demographique (INED), Mortality, Health and Epidemiology Unit, Paris, France; 3 Institut de Recherche pour le Developpement (IRD), UMI 174/PHPT, Chiang Mai, Thailand; 4 Unitat d’Infectologia, Servei de Pediatria, Hospital Sant Joan de Deu, Universitat de Barcelona, Barcelona, Spain; 5 Hopital St Pierre, Brussels, Belgium; 6 St George’s Healthcare NHS Trust, London, United Kingdom; 7 Department of Health Sciences, Pediatric Unit, University of Florence, Florence, Italy; 8 Department of Pediatric Infectious Diseases, Emma Children’s Hospital, Academic Medical Center, Amsterdam, the Netherlands; 9 Centro Hospitalar do Porto, Porto, Portugal; 10 Hospital Doce de Octubre, Madrid, Spain; 11 Hospital General Universitario "Gregorio Marañón", Madrid, Spain; 12 Institut National de la Santé et de la Recherche (INSERM), Paris, France; 13 University Hospital Frankfurt, Department of Paediatrics, Goethe University, Frankfurt, Germany; 14 University of Athens Medical School, Athens, Greece; 15 Hospital de Santa Maria, Lisbon, Portugal; 16 Karolinska University Hospital, Stockholm, Sweden; 17 Paediatric European Network for the Treatment of AIDS (PENTA), Padova, Italy; 18 UCL Great Ormond Street Institute of Child Health, London, United Kingdom; 19 Medical University of Warsaw, Hospital of Infectious Diseases, Warsaw, Poland; 20 Republican Hospital of Infectious Diseases, St Petersburg, Russia; 21 Hopitaux Universitaires de Genève, Genève, Switzerland; 22 Chiang Rai Prachanukroh Hospital, Chiang Rai, Thailand; 23 Department of Pediatrics, Phayao Provincial Hospital, Phayao, Thailand; 24 Shupyk National Medical Academy of Postgraduate Education, Kiev, Ukraine; 25 Perinatal Prevention of AIDS Initiative, Odessa, Ukraine; 26 Victor Babes Hospital, Bucharest, Romania; San Francisco General Hospital, UNITED STATES

## Abstract

**Background:**

Published estimates of mortality and progression to AIDS as children with HIV approach adulthood are limited. We describe rates and risk factors for death and AIDS-defining events in children and adolescents after initiation of combination antiretroviral therapy (cART) in 17 middle- and high-income countries, including some in Western and Central Europe (W&CE), Eastern Europe (Russia and Ukraine), and Thailand.

**Methods and findings:**

Children with perinatal HIV aged <18 years initiating cART were followed until their 21st birthday, transfer to adult care, death, loss to follow-up, or last visit up until 31 December 2013. Rates of death and first AIDS-defining events were calculated. Baseline and time-updated risk factors for early/late (≤/>6 months of cART) death and progression to AIDS were assessed. Of 3,526 children included, 32% were from the United Kingdom or Ireland, 30% from elsewhere in W&CE, 18% from Russia or Ukraine, and 20% from Thailand. At cART initiation, median age was 5.2 (IQR 1.4–9.3) years; 35% of children aged <5 years had a CD4 lymphocyte percentage <15% in 1997–2003, which fell to 15% of children in 2011 onwards (*p* < 0.001). Similarly, 53% and 18% of children ≥5 years had a CD4 count <200 cells/mm^3^ in 1997–2003 and in 2011 onwards, respectively (*p* < 0.001). Median follow-up was 5.6 (2.9–8.7) years. Of 94 deaths and 237 first AIDS-defining events, 43 (46%) and 100 (42%) were within 6 months of initiating cART, respectively. Multivariable predictors of early death were: being in the first year of life; residence in Russia, Ukraine, or Thailand; AIDS at cART start; initiating cART on a nonnucleoside reverse transcriptase inhibitor (NNRTI)-based regimen; severe immune suppression; and low BMI-for-age z-score. Current severe immune suppression, low current BMI-for-age z-score, and current viral load >400 c/mL predicted late death. Predictors of early and late progression to AIDS were similar. Study limitations include incomplete recording of US Centers for Disease Control (CDC) disease stage B events and serious adverse events in some countries; events that were distributed over a long time period, and that we lacked power to analyse trends in patterns and causes of death over time.

**Conclusions:**

In our study, 3,526 children and adolescents with perinatal HIV infection initiated antiretroviral therapy (ART) in countries in Europe and Thailand. We observed that over 40% of deaths occurred ≤6 months after cART initiation. Greater early mortality risk in infants, as compared to older children, and in Russia, Ukraine, or Thailand as compared to W&CE, raises concern. Current severe immune suppression, being underweight, and unsuppressed viral load were associated with a higher risk of death at >6 months after initiation of cART.

## Abstract 2

In a multi-country cohort study, Ali Judd and colleagues study morbidity, mortality, and relevant risk factors in children and adolescents with HIV infection receiving antiretroviral treatment.

## Author summary

**Why was this study done?:**

- Adolescence has been highlighted as an urgent global priority for health by a diverse range of stakeholders.
- HIV/AIDS is one of the top 10 leading causes of death in adolescents globally.
- Estimates of mortality and progression to AIDS as children approach adulthood are limited.

**What did the research do and find?:**

- We investigated long-term risk of death and AIDS-defining events in children and adolescents with perinatal HIV across high- and middle-income country settings in Western and Central Europe, Eastern Europe (Russia, Ukraine), and Thailand.
- In our study, in 3,526 patients across 17 countries, median age at cART initiation was 5.2 years, and median duration of follow-up was 5.6 years, with 9,228 person-years of follow-up in those aged ≥10 years and 20,574 person-years overall.
- We found that there was a higher risk of mortality in very young children, in Eastern European countries and Thailand, and in patients with current severe immune suppression and low BMI.

**What do these findings mean?:**

- We describe outcomes for some of the first generation of children to survive perinatal HIV and reach adolescence.
- Our findings, together with other available evidence, suggest an ongoing need for intensive monitoring of very young children with HIV infection, those with severe immunosuppression, and those with a lower BMI than that expected for their age.

## Introduction

Studies have reported declining mortality since the introduction of combination antiretroviral therapy (cART) about 20 years ago, both in adults and children [1–3]. For example, in the United Kingdom (UK) and Irish nationwide paediatric cohort, among 1,441 children with median age 9 years at last follow-up, mortality rates declined from 8.2 per 100 person-years before 1996 to 0.6 in 2003–2006 [1]. In an Italian multicentre study, survival of children with perinatal HIV significantly improved between 1996 and 1998, following the introduction of cART [4]. Similarly, in a United States cohort of 3,553 children with median age at enrolment of 6 years and median follow-up of 5 years, mortality rates declined from 7.2 to 0.8 per 100 person-years between 1994 and 2000 and remained relatively stable to the end of 2006 [2]. Mortality rates in adults have shown a similar trend, and life expectancy among those with a CD4 count ≥500 cells/mm^3^ is now thought to be close to that of the general population [5].

However, AIDS is now a top 10 leading cause of death in adolescents globally [6], and evidence suggests worsening outcomes as children with HIV grow up and transition to adult care. European data from individual cohorts suggest that young people with perinatal HIV infection have a higher risk of treatment failure [7–10], care disengagement [11], and death [12] compared to those who acquire HIV in adulthood and children with other routes of transmission. Thus, there is the need for vigilance to ensure that health outcomes in this population who have survived paediatric HIV are maximised as they enter adulthood.

Longitudinal cohort studies are well placed to measure changes in mortality rates over time. However, due to relatively limited numbers of children with perinatal HIV infection in some European countries [13], pooling of data across cohorts is the only practical way of obtaining reliable mortality rates across the European region. The European Pregnancy and Paediatric HIV Cohort Collaboration (EPPICC) is a network of 19 cohorts across 17 countries in Western and Central Europe (W&CE), Eastern Europe (Russia, Ukraine), and Asia (Thailand). In this study, data from these cohorts were used to describe rates and risk factors for mortality and AIDS-defining events in perinatally HIV-infected children and adolescents after initiating cART.

## Methods

This study was carried out in accordance with the EPPICC Paediatric merger 2014 SOP and the project-specific Concept Sheet (S1 Text). Nineteen cohorts across 17 countries contributed to individual patient data (see S2 Text: “Writing Group members and collaborating cohorts” for list of collaborators). Children were included in this analysis if they had perinatal HIV infection and initiated cART (defined as a ≥3 drug, ≥2 class regimen [excluding unboosted PIs] or ≥3 non-nucleoside reverse transcriptase inhibitor [NNRTI]-only regimen, including abacavir) after 1996 (with no prior antiretroviral therapy [ART] use), aged <18 years. They were at risk from cART initiation until their censor date, defined as the earliest of 21st birthday, last visit in paediatric care, death, or loss to follow-up (as defined by each cohort), with data available until 31 December 2013. Demographic, clinical, laboratory, and treatment-related data from routine clinic visits (typically every 3–6 months) were pooled electronically using a modified HICDEP protocol (www.hicdep.org). Pooled data were subjected to a battery of consistency checks. Data on all children at participating clinics were included; data are pseudo-anonymised, and therefore individual informed consent was not obtained. All cohorts received approval from local and/or national ethical committees. For example, the UK/Ireland CHIPS cohort had approval from the London Central Research Ethics Committee.

Causes of death, along with CD4 and clinical event data, were reviewed by the Project Team and confirmed by the reporting clinician. Cause of death was coded using the International Classification of Diseases version 10 and categorised into 4 groups: HIV-related infectious causes, other HIV-related causes, deaths not directly related to HIV, and those with unknown cause. AIDS events were considered to be reliably reported, as they are an important clinical indicator of disease progression for children in routine care.

### Statistical methods

Rates of death and first AIDS-defining event were calculated per 100,000 person-years. Rates were summarised overall, within 6 months of ART initiation (‘early’; children were at risk from ART initiation to ART initiation plus 6 months), and after 6 months of ART (‘late’; at risk from ART initiation plus 6 months to their censor date), and rates of first AIDS-defining event were additionally summarised by event. Early deaths and AIDS-defining events were summarised separately, as they were likely to be related to late presentation/initiation of cART. AIDS-defining events were classified according to the US Centers for Disease Control and Prevention 2014 surveillance criteria [14]. Cohorts reported date of AIDS diagnosis as the date of the child’s earliest WHO stage 3/4 or US Centers for Disease Control (CDC) stage C event. Continuous variables were compared using Wilcoxon’s rank-sum test and categorical variables using a chi-squared test.

Risk factors for early death, late death, early first AIDS-defining event, and late first AIDS-defining event were assessed using univariable and multivariable proportional hazard models with inverse-probability-of-censoring weights. The probability of being censored was estimated using logistic regression (including all factors listed below) to account for informative censoring in those lost to follow-up. Weights were calculated as the reciprocal of these estimated probabilities. Association with the following factors at cART initiation were considered: age (continuous), sex, ethnicity (black African, Asian, other, unknown), year of birth (continuous), born abroad (versus in country of cohort), country group (Eastern Europe and Thailand [EE&T] [Russia/Ukraine/Thailand]) versus W&CE (all others), previous AIDS diagnosis (yes, no), year of cART initiation (continuous), initial regimen (NNRTI-based, other), World Health Organization (WHO) severe immune suppression for age (defined as a CD4 lymphocyte percentage [CD4%] <25% for children <1 year of age, <20% for children aged 1–3 years, <15% for children aged 3–5 years, and <200 cells/mm^3^ or <15% for children aged ≥5 years [15]), viral load (≤100,000 c/mL, >100,000 c/mL), and BMI-for-age z-score (>0, 0–−3, <−3, calculated using WHO child growth standards, 2007).

Time-updated factors considered for late death and first AIDS event were age, severe immune suppression for age, HIV viral load, BMI-for-age z-score, and proportion of time suppressed since cART initiation (≥80% versus <80%, as a proxy for adequate adherence). Values for time-updated factors based on clinical/laboratory measurements were considered to occur on the date recorded and were carried forward for up to 6 months (if no subsequent measurement was recorded), after which they were considered to be unknown. Individuals with missing data for any given factor were classified as missing, with a separate level of the variable (the missing indicator method).

Nonlinear effects of continuous variables were explored using natural cubic splines with 5 knots, and in final models, where appropriate, statistically significant nonlinearity was represented by piecewise linear functions. All characteristics (excluding those that were highly correlated) were included in a multivariable model. Covariate interactions between country group, calendar year of ART initiation, and any other statistically significant (*p* < 0.05) characteristics were considered and the proportional hazards assumption assessed.

BMI-for-age z-score was chosen rather than weight-for-age z-score, as WHO has normative data across all ages for BMI for age (WHO reference data for weight are only available for children aged <10 years), and to minimise differences due to variation in height for age between ethnic groups in the general population. A sensitivity analysis used weight-for-age z-score based on UK normative data for Western, Central, and Eastern European cohorts and Thai normative data for the Thai cohort. Analyses investigating rates and risk factors for early and late AIDS events separately were not prespecified but conducted following discussion of the results from a combined analysis investigating overall rates of AIDS events. Statistical analyses were performed using Stata version 14.2 (Stata Corporation, College Station, Texas). STROBE recommendations were followed (S1 STROBE Checklist).

## Results

Of 3,953 HIV-infected, ART-naïve children in EPPICC at the time of initiating cART, 3,526 (89%) acquired HIV perinatally and were eligible for this analysis; 1,124 (32%) were from the UK or Ireland, 700 (20%) from Thailand, 508 (14%) from Ukraine, and 1,194 (34%) from elsewhere across Europe (Table 1). The median age at cART initiation for the 3,526 participants was 5.2 (IQR 1.4–9.3) years, and the median year of cART initiation was 2006 (2003–2009). Median duration of follow-up was 5.6 (2.9–8.7) years, with 20,574 person-years of follow-up overall (of which 4,531 were of participants aged <5 years, 6,836 aged 5–<10 years, 6,577 aged 10–<15 years, 2,139 aged 15–<18 years, and 512 aged ≥18 years).

**Table 1:** Characteristics of children overall, and by timing of death, for those who died.

Characteristics		Overall (*N* = 3,526)	Died ≤6 months after cART initiation (*N* = 43)	Died >6 months after cART initiation (*N* = 51)
		*n* (%) or median [IQR]		
*Sociodemographics*:				
Country of cohort	Belgium	71 (2)	0	1 (2)
	France	124 (4)	0	1 (2)
	Germany	17 (0.5)	0	0
	Greece	20 (1)	0	0
	Italy	206 (6)	1 (2)	1 (2)
	the Netherlands	160 (5)	0	0
	Poland	51 (1)	1 (2)	0
	Portugal	28 (1)	0	0
	Romania	28 (1)	0	0
	Russia	132 (4)	1 (2)	2 (3)
	Spain	258 (7)	0	1 (2)
	Sweden	64 (2)	0	0
	Switzerland	35 (1)	0	1 (2)
	Thailand	700 (20)	25 (58)	29 (57)
	UK/Ireland	1,124 (32)	12 (28)	13 (25)
	Ukraine	508 (14)	3 (7)	2 (4)
Sex	Male	1,670 (47)	18 (42)	21 (41)
	Female	1,856 (53)	25 (58)	30 (59)
Ethnic group	Black African	1,176 (33)	10 (23)	13 (25)
	Asian	723 (21)	25 (58)	29 (57)
	Other^†^	982 (28)	6 (14)	5 (10)
	Unknown	645 (18)	2 (5)	4 (8)
Born abroad		1,081 (32)	8 (23)	9 (20)
Year of birth	<2000	1,632 (46)	25 (58)	39 (76)
	≥2000	1,894 (54)	18 (42)	12 (24)
*Characteristics at HIV diagnosis*:				
Age (years)		2.7 [0.7–7.4]	5.6 [0.5–8.0]	7.9 [2.2–10.3]
CD4% (*n* = 1,580, 23, 20)		19 [10–29]	11 [1–22]	5 [1–16]
*Characteristics at cART initiation*:				
Age (years)		5.2 [1.4–9.3]	6.2 [0.6–9.9]	8.1 [4.6–10.8]
Age ≥5 years		1,804 (51)	26 (60)	35 (69)
AIDS diagnosis		663 (19)	26 (60)	14 (27)
Calendar year	1997–2003	973 (28)	19 (44)	30 (59)
	2004–2007	1,264 (36)	13 (30)	16 (31)
	≥2008	1,289 (37)	11 (26)	5 (10)
Initial cART regimen	NNRTI-based	2,227 (63)	36 (84)	43 (84)
	PI-based	1,140 (32)	7 (16)	6 (12)
	Other	159 (4)	0	2 (4)
CD4% (*n* = 2,677, 41, 34)		16 [9–25]	5 [1–14]	5 [1–15]
CD4 count (age ≥5 years) (*n* = 1,436, 25, 22)		242 [85–410]	14 [7–29]	55 [10–134]
Immune suppression for age (*n* = 2,719, 42, 35)*	Not severe	1,228 (45)	6 (14)	5 (14)
	Severe	1,491 (55)	36 (86)	30 (86)
Viral load (log_10_ c/mL) (*n* = 2,481, 33, 30)		5.0 [4.4–5.7]	5.5 [4.9–5.9]	5.3 [4.3–5.7]
Height-for-age z-score (*n* = 1,877, 30, 27)		−1.2 [−2.3–−0.2]	−3.4 [−4.4–−2.1]	−2.3 [−3.4–−1.8]
BMI-for-age z-score (*n* = 1,872, 29, 27)		−0.2 [−1.1–0.8]	−2.8 [−3.8–−0.8]	−1.5 [−2.7–−0.3]

*See Methods for definition.

^†^ Other ethnicity: 950 children reported as white, 23 Hispanic, 8 mixed, 1 indigenous.

cART, combination antiretroviral therapy; CD4%, CD4 lymphocyte percentage; NNRTI; nonnucleoside reverse transcriptase inhibitor; PI, protease inhibitor.

**Fig 1:**
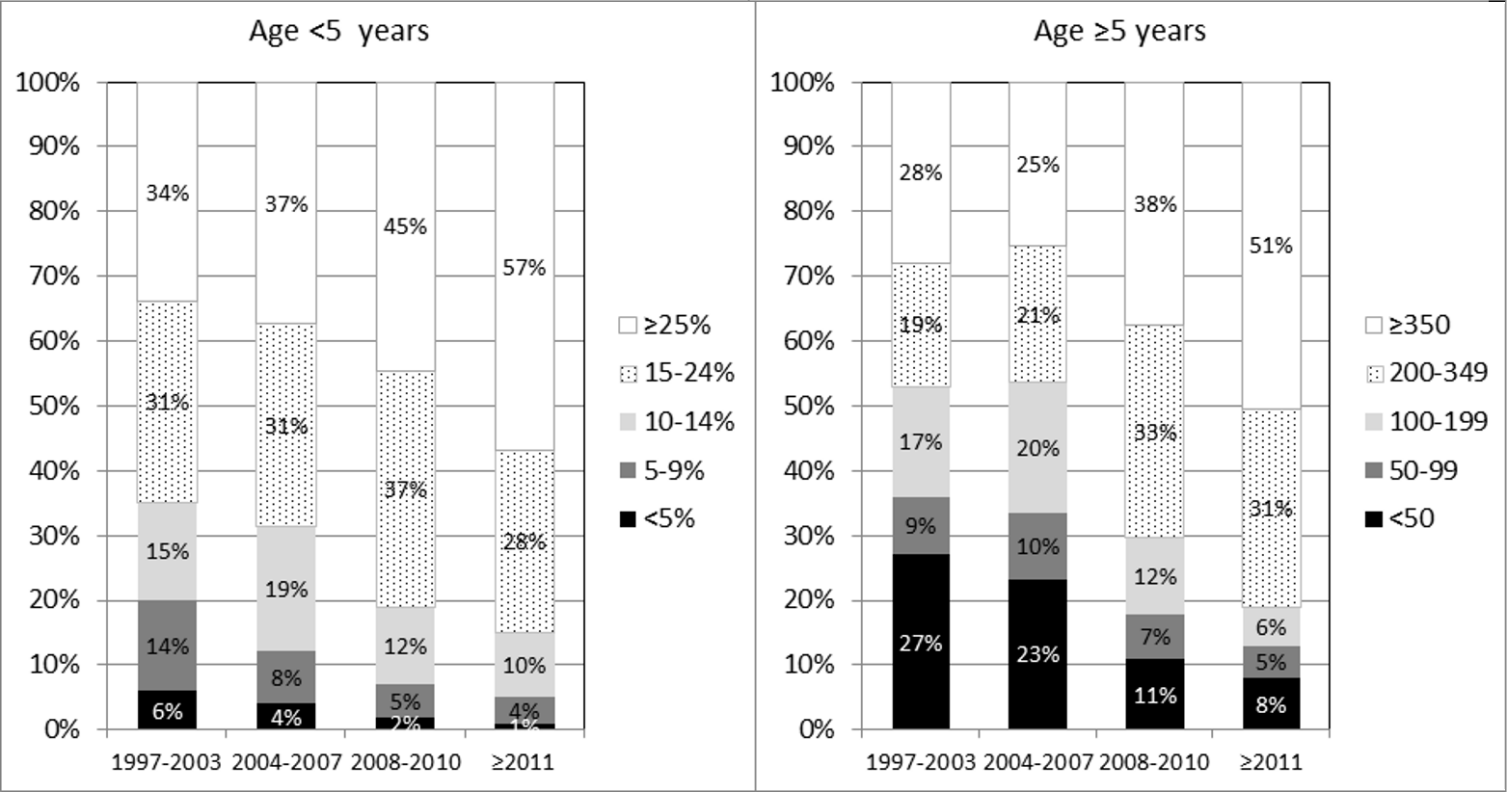
CD4% (left panel) and count (right panel) at initiation of cART, by age group and calendar period. cART, combination antiretroviral therapy; CD4%, CD4 lymphocyte percentage.

At cART initiation, 1,491/2,719 (55%) children overall had severe immune suppression for their age (466/555 [84%] for Thailand) and 663 (19%) had a previous AIDS diagnosis. The proportion of children aged <5 years at ART initiation who had a CD4% less than 15% declined over the study period, from 35% (134/381) in 1997–2003 to 15% (24/157) from 2011 onwards (*p* < 0.001) (Fig 1), and similarly, the proportion aged ≥5 years with a CD4 count <200 cells/mm^3^ at cART initiation declined from 53% (194/365) to 18% (36/196) (*p* < 0.001).

By the end of the study period, 94 (3%) children had died, 453 (13%) had been lost to follow-up, 305 (9%) had transferred to adult care, 314 (9%) had dropped out for other reasons (including having moved to another clinic or withdrawn consent), and 2,322 (66%) were still in follow-up. Among those still in paediatric care, the median age at last visit was 11.6 (7.6–15.0) years.

### Causes of death

**Fig 2:**
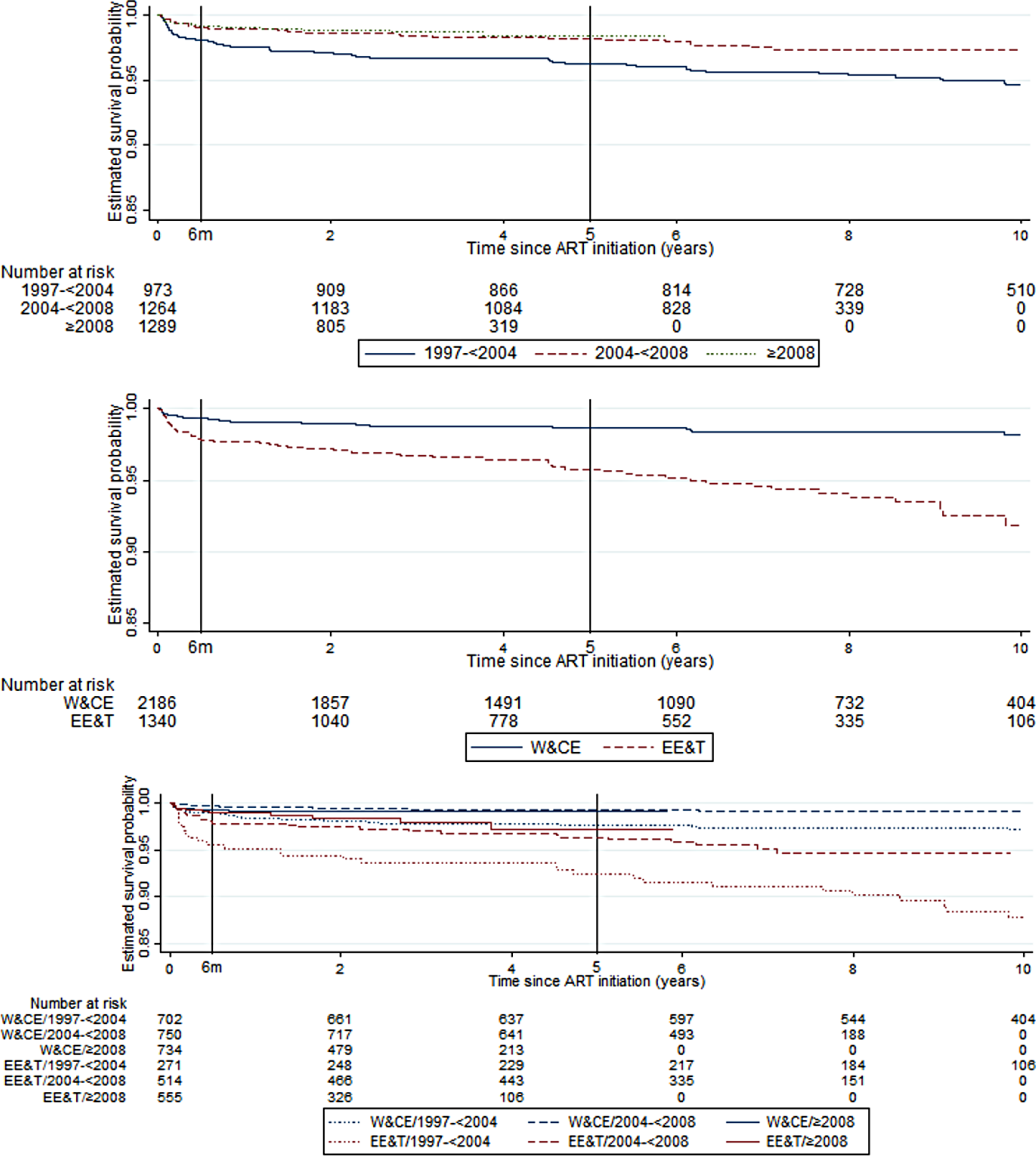
Kaplan-Meier survival curve for time from cART initiation to death (A) by calendar period of cART initiation, (B) by country group*, and (C) by calendar period and country group. ART, antiretroviral therapy; cART, combination antiretroviral therapy; EE&T, Eastern Europe and Thailand; W&CE, Western and Central Europe.

**Fig 3:**
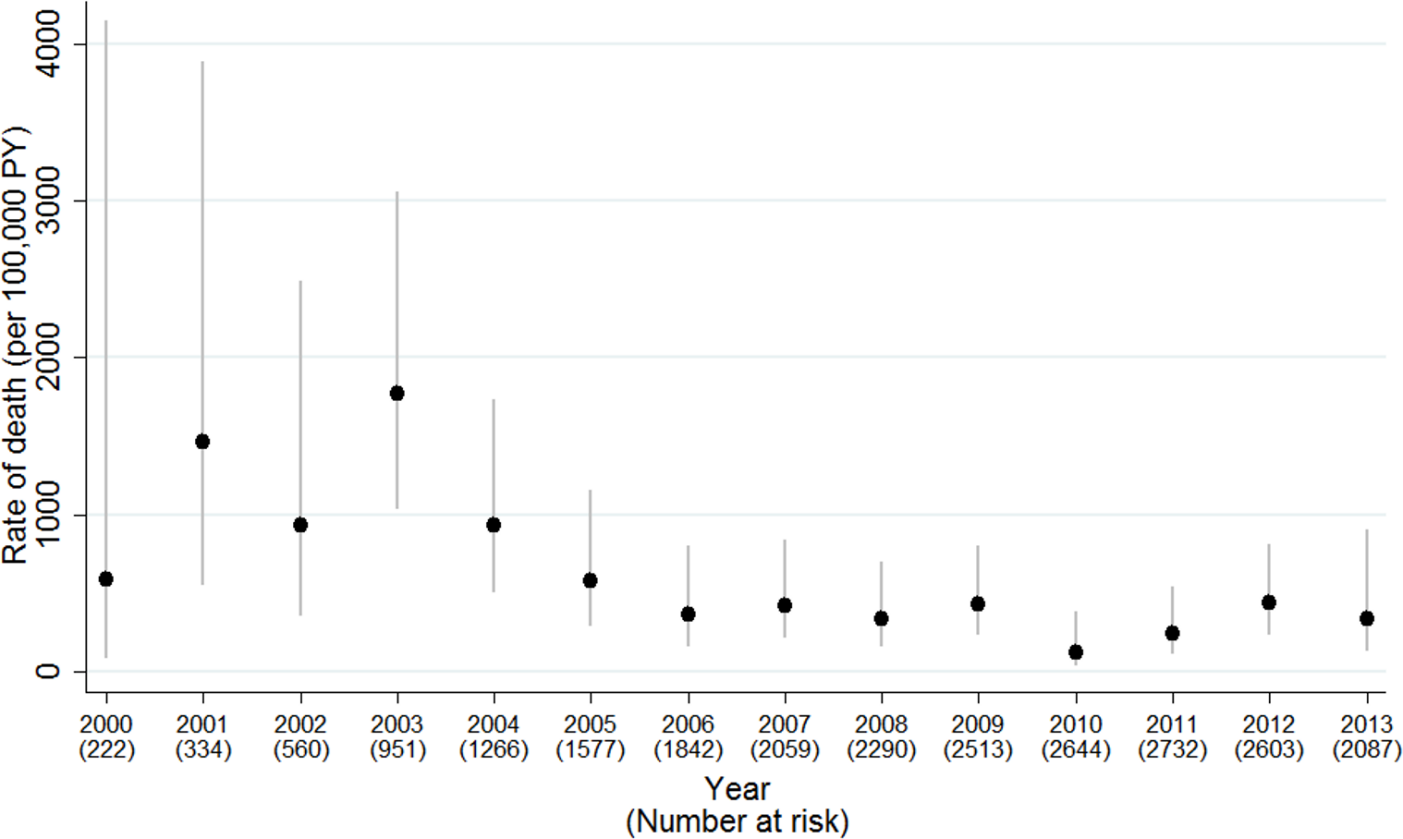
Mortality rates (95% CI) by calendar year of follow-up, 2000–2013.

There were 94 deaths, of which 43 (46%) were within 6 months of cART initiation. Among the early deaths, 37% (16/43) occurred within 1 month and 79% (34/43) within 3 months of cART start. The crude mortality rate was 2,502 (95% CI 1,856–3,374) per 100,000 person-years in the first 6 months of treatment and 270 (206–356) thereafter. Fig 2 shows the decreasing risk of death after the first 6 months of cART, in all calendar periods and country groups. The probability of survival to 5 years after initiating cART was 97.6% (97.0%–98.1%) overall and improved with calendar period from 96.2% (94.8%–97.2%) in 1997–2004 to 98.4% (97.2%–99.1%) for 2008 onwards. Overall, it was 98.7% (98.0%–99.1%) in W&CE countries and 95.8% (94.4%–96.8%) in EE&T countries. The mortality rate was highest in earlier calendar years, peaking at 1,773 (95% CI 1,030–3,054) per 100,000 person-years in 2003 and decreasing to 360 in 2006, after which it was relatively stable at between 122 and 435 cases per 100,000 person-years (Fig 3), with a similar trend for deaths both before and after 6 months of cART (S1 Fig). The peak in mortality rate in 2003 coincided with the introduction of cART in Thailand, and Thailand accounted for over half of the deaths in the periods for both early and late deaths (Table 1).

The median age at death was 9.5 (IQR 3.3–13.9) years; those who died within 6 months of cART initiation were younger than those who died after 6 months (median age 6.5 versus 12.8, *p* < 0.001). Among those who died after 6 months of cART, the median (IQR) viral load was 9,393 (400–107,654), CD4% was 6% (2%–24%), and CD4 count (among children aged ≥5 years, *n* = 26) was 68 (9–304) cells/mm^3^. Twelve (24%) children with late deaths were not taking cART at the time of death, and a further 4 (8%) had a treatment interruption of >30 days in the 6 months prior to death.

Overall, 58 (64%) deaths were due to HIV-related infections, predominantly bacterial infection and/or sepsis (Table 2), and these accounted for 76% (31/41 with cause available) of early deaths and 55% (27/49) of late deaths (*p* = 0.043). Twenty-four deaths (27%) were from other HIV-related causes (22% versus 31% early versus late, *p* = 0.355), 8 (9%) were not directly related to HIV (2% versus 14%, *p* = 0.049), and 4 causes were unknown. Over the calendar periods 2000–2004, 2005–2008, and 2009 onwards, the proportions of deaths caused by HIV-related infections (19/30: 63%, 21/29: 72%, and 18/31: 58%, respectively) and other HIV-related causes (10/30: 33%, 3/29: 10%, and 11/31: 35%, respectively) did not significantly change (*p* = 0.504 and *p* = 0.053, respectively). Overall, 45 (50%) deaths were AIDS related (59% of early deaths versus 43% later, *p* = 0.138), with no discernible trend by calendar year period (8/30: 27%, 15/29: 52%, 11/31: 35%) or country group (EE&T: 30/59, 51%; W&CE: 15/31, 48%; *p* = 0.824). Twenty-five (27%) children who died did not have an AIDS-defining illness at the time of their death; their most common cause of death was bacterial infection/sepsis.

**Table 2:** Causes of death for children who died, overall, and by timing of death.

Category	Cause	Total (*N* = 94)	Time since cART initiation	
			≤6 months (*N* = 43)	>6 months (*N* = 51)
		*n* (%)		
HIV-related infections	Bacterial infection and/or sepsis	26	15	11
	(Bacterial pneumonia/pneumosepsis)*	(13)	(9)	(4)
	Cryptococcosis	6	1	5
	PJP	6	4	2
	Tuberculosis	3	2	1
	Nontuberculous mycobacteria	4	1	3
	CMV	3	2	1
	PJP + CMV	3	2	1
	Cerebral toxoplasmosis	2	2	0
	Unspecified	1	0	1
	Other^1^	4	2	2
	*Total HIV related*	*58 (64%)*	*31 (76%)*	*27 (55%)*
Other HIV related	Wasting syndrome	8	3	5
	Heart failure/cardiomyopathy	6	4	2
	Malignant neoplasm^2^	4	1	3
	Haemorrhage^3^	3	0	3
	Encephalopathy	1	1	0
	Lactic acidosis	1	0	1
	Myelodysplasia	1	0	1
	*Total other HIV related*	*24 (27%)*	*9 (22%)*	*15 (31%)*
Not directly HIV related	Haemorrhage^4^	2	1	1
	Suicide	2	0	2
	Accident	1	0	1
	Atrioventricular block	1	0	1
	Congenital malformation	1	0	1
	Diabetes mellitus	1	0	1
	*Total not directly HIV related*	*8 (9%)*	*1 (2%)*	*7 (14%)*
Unknown	*Total unknown*	*4*	*2*	*2*

*Subset of previous category.

^1^ 1 cysticercosis of the central nervous system, 1 geotrichosis, 1 fungal pneumonia, 1 progressive multifocal leukoencephalopathy.

^2^ 1 Kaposi sarcoma, 1 non-Hodgkin’s lymphoma, 2 unspecified.

^3^ 2 cerebral haemorrhages, categorised as HIV-related as they occurred with other HIV-related conditions, such as chronic thrombocytopenia, that could lead to haemorrhage.

^4^ 1 cerebral, 1 haematemesis. No other HIV-related conditions that could have explained haemorrhage were reported.

cART, combination antiretroviral therapy; CMV, cytomegalovirus; PJP, *Pneumocystis jirovecii* pneumonia.

Of the 51 late deaths, 21 (41%) were in adolescents aged ≥14 years. Median ages at HIV diagnosis and cART initiation of these 21 children were 9.9 (IQR 8.5–11.3) and 10.8 (8.7–11.9) years, respectively, and median duration of cART was 6.2 (4.5–7.6) years. Thirteen (62%) of these deaths were due to an HIV-related infection, 6 to other HIV-related causes, and 2 were unrelated to HIV.

### Risk factors for death

In multivariable analysis, being in the first year of life, EE&T country group, AIDS diagnosis at cART initiation, initiating cART on an NNRTI-based regimen (versus any other), severe immune suppression for age, and low BMI-for-age z-score were associated with increased risk of early death (Table 3). Sensitivity analysis replacing BMI-for-age z-score with weight-for-age z-score resulted in similar estimates (S1 Table).

**Table 3:** Rates and baseline risk factors for death within 6 months of cART initiation.

Baseline characteristics at initiation of cART		*N* deaths	Rate per 100,000 PY (95% CI)	Univariable			Multivariable		
				HR	95% CI	*p*	HR	95% CI	*p*
Sex	Male	18	2,208 (1,391–3,504)	0.80	0.44–1.47	0.469	0.86	0.45–1.64	0.656
	Female	25	2,769 (1,871–4,098)	1.00	-		1.00	-	
Age (per year increase)	<2 years	16	3,143 (1,925–5,130)	0.43	0.25–0.73	0.024	0.31	0.13–0.73	0.048
	≥2–<8 years	12	1,852 (1,052–3,261)	1.27	1.04–1.54		1.23	0.97–1.56	
	≥8 years	15	2,674 (1,612–4,435)	0.93	0.76–1.13		0.94	0.72–1.23	
Year of birth (per year increase)		<2003	31	2,777 (1,953–3,949)	0.93	0.87–1.00	0.012	-			
		≥2003–<2007	4	1,208 (453–3,219)	0.87	0.64–1.19					
		≥2007	8	2,954 (1,477–5,906)	1.55	1.12–2.14					
Place of birth	Within country	27	2,435 (1,670–3,551)	1.00	-	<0.001	-			
	Abroad	8	1,507 (754–3,013)	0.62	0.28–1.37					
	Unknown	8	10,172 (5,087–20,340)	4.19	1.90–9.25					
Ethnicity	Black African	10	1,732 (932–3,220)	0.24	0.12–0.50	<0.001	-			
	Asian	25	7,215 (4,875–10,677)	1.00	-					
	Other	6	1,258 (565–2,800)	0.17	0.07–0.43					
	Unknown	2	630 (158–2,520)	0.09	0.02–0.37					
Country group	W&CE	14	1,304 (772–2,201)	0.29	0.15–0.55	<0.001	0.39	0.17–0.89	0.025
	EE&T	29	4,501 (3,128–6,477)	1.00	-		1.00	-	
AIDS diagnosis	No AIDS	17	1,214 (755–1,953)	1.00	-	<0.001	1.00	-	<0.001
	AIDS	26	8,183 (5,571–12,018)	6.73	3.65–12.43		3.91	2.00–7.64	
Year of cART initiation (per year increase)	-	-	0.65	0.43–0.96	0.032	0.85	0.54–1.33	0.469
Initial regimen	NNRTI-based	36	3,312 (2,389–4,592)	1.00	-	0.008	1.00	-	0.057
	PI-based/other	7	1,109 (529–2,326)	0.33	0.15–0.75		0.43	0.18–1.03	
Immune suppression for age	Not severe	6	1,002 (450–2,230)	0.20	0.08–0.47	<0.001	0.37	0.15–0.91	0.004
	Severe	36	5,006 (3,611–6,940)	1.00	-		1.00	-	
	Unknown	1	250 (35–1,774)	0.05	0.01–0.37		0.08	0.01–0.55	
Viral load (c/mL)	≤100,000	12	2,083 (1,183–3,667)	0.63	0.31–1.27	0.260	0.89	0.41–1.92	0.955
	>100,000	21	3,349 (2,183–5,136)	1.00	-		1.00	-	
	Unknown	10	1,942 (1,045–3,610)	0.59	0.28–1.24		0.95	0.41–2.21	
BMI-for-age z-score	>0	5	1,193 (497–2,866)	0.44	0.15–1.24	<0.001	1.02	0.32–3.22	0.006
	−3–0	12	2,726 (1,548–4,800)	1.00	-		1.00	-	
	<−3	12	26,209 (14,884–46,150)	9.47	4.23–21.19		4.64	1.90–11.34	
	Unknown	14	1,722 (1,020–2907)	0.64	0.29–1.38		1.30	0.57–3.44	

Notes: The following variables were excluded from the multivariable model due to correlation: year of birth (with age and, also, year of cART initiation); place of birth (with country group); ethnicity (with country group).

cART, combination antiretroviral therapy; EE&T, Eastern Europe and Thailand; HR, hazard ratio; NNRTI, nonnucleoside reverse transcriptase inhibitor; PI, protease inhibitor; PY, person years; W&CE, Western and Central Europe.

Rates and risk factors for death after 6 months of cART are shown in Table 4. In multivariable analysis, no baseline characteristics were strongly associated with higher risk of late death. Time-updated characteristics associated with higher risk of death were severe immune suppression for age, current viral load >400 c/mL, and low current BMI-for-age z-score. There was a nonsignificantly elevated risk of late death in EE&T (*p* = 0.073). After adjustment for other factors, year of cART initiation was no longer associated with early or late death.

**Table 4:** Rates and risk factors (baseline and time updated) for death after 6 months of cART.

Baseline characteristics at initiation of cART		*N* deaths	Rate per 100,000 PY (95% CI)	Univariable			Multivariable		
				HR	95% CI	*p*	HR	95% CI	*p*
Sex	Male	21	232 (151–356)	0.74	0.42–1.29	0.290	0.91	0.51–1.59	0.731
	Female	30	306 (214–437)	1.00	-		1.00	-	
Age (per year increase)	<2 years	9	155 (81–298)	0.80	0.47, 1.38	<0.001	0.67	0.34–1.31	0.309
	≥2 years	42	322 (238–435)	1.18	1.09–1.27		1.08	0.97–1.20	
Year of birth (per year increase)	<1995	20	567 (366–878)	0.98	0.82–1.17	<0.001	-		
	≥1995–<2000	19	270 (172–423)	0.72	0.58–0.89				
	≥2000	12	145 (82–255)	1.06	0.88–1.27				
Place of birth	Within country	37	302 (219–417)	1.00	-	0.066	-		
	Abroad	9	159 (83–305)	0.50	0.24–1.04				
	Unknown	5	531 (221–1277)	1.71	0.67–4.38				
Ethnicity	Black African	35	7,447 (5,347–10,372)	0.56	0.35–0.90	0.012	-		
	Asian	35	13,250 (9,514–18,455)	1.00	-				
	Other	25	6,573 (4,441–9,727)	0.50	0.30–0.83				
	Unknown	15	5,899 (3,557–9,786)	0.45	0.24–0.82				
Country group	W&CE	18	142 (90–226)	0.25	0.13, 0.45	<0.001	0.48	0.22–1.07	0.073
	EE&T	33	531 (377–747)	1.00	-		1.00	-	
AIDS diagnosis	No AIDS	37	245 (178–338)	1.00	-	0.218	1.00	-	0.169
	AIDS	14	372 (221–629)	1.48	0.79–2.75		1.53	0.84–2.80	
Year of cART initiation (per year increase)		-	-	0.62	0.39–0.99	0.044	0.62	0.35–1.91	0.091
Initial regimen	NNRTI-based	43	328 (243–442)	1.00	-	0.018	1.00	-	0.637
	PI-based/other	8	139 (70–279)	0.40	0.19–0.86		0.82	0.35–1.91	
Immune suppression for age	Not severe	5	85 (35–204)	0.26	0.10–0.68	0.019	0.77	0.24–2.49	0.875
	Severe	30	357 (250–511)	1.00	-		1.00	-	
	Unknown	16	349 (214–570)	1.00	0.54–1.84		1.02	0.42–2.45	
VL (c/mL)	≤100,000	13	214 (124–369)	0.94	0.45–1.96	0.247	0.72	0.36–1.44	0.615
	>100,000	17	241 (150–387)	1.00	-		1.00	-	
	Unknown	21	366 (239–562)	1.57	0.82–2.99		0.80	0.37–1.77	
BMI-for-age z-score	>0	2	42 (10–166)	0.09	0.02–0.38	0.007	0.18	0.04–0.76	0.078
	−3–0	22	440 (289–668)	1.00	-		1.00	-	
	<−3	3	556 (179–1,723)	1.20	0.35–4.10		0.46	0.14–1.51	
	Unknown	24	282 (189–421)	0.63	0.35–1.12		0.71	0.27–1.83	
**Time updated characteristics**									
Age (per year increase)	<5 years	7	186 (89–390)	0.65	0.45–0.95	<0.001	-		
	≥5 years	44	292 (217–292)	1.22	1.13–1.32				
Immune suppression for age	Not severe	9	67 (35–128)	0.02	0.01–0.05	<0.001	0.07	0.02–0.17	<0.001
	Severe	30	2,617 (1,830–3,743)	1.00	-		1.00	-	
	Unknown	12	285 (162–502)	0.10	0.05–0.20		0.19	0.05–0.73	
VL (c/mL)	≤400	12	102 (58–179)	0.10	0.05–0.21	<0.001	0.32	0.13–0.78	0.028
	>400	25	958 (647–1,418)	1.00	-		1.00	-	
	Unknown	14	315 (187–532)	0.33	0.17–0.65		0.63	0.18–2.27	
% time since cART initiation with VL ≤400 c/mL	<80%	30	477 (334–682)	1.00	-	<0.001	-		
	≥80%	7	86 (41–181)	0.18	0.08–0.41				
	Unknown	14	315 (187–532)	0.67	0.35–1.28				
BMI-for-age z-score	>0	8	130 (65–260)	0.55	0.23–1.30	<0.001	1.14	0.43–2.97	<0.001
	−3–0	14	230 (136–388)	1.00	-		1.00	-	
	<−3	12	9,006 (5,115–15,859)	42.27	19.30–92.57		17.37	6.31–47.82	
	Unknown	17	262 (163–421)	1.12	0.54–2.32		1.61	0.59–4.44	

Notes: The following variables were excluded from the multivariable model due to correlation: year of birth (with age and also year of cART initiation); place of birth (with country group); ethnicity (with country group); time-updated age (with age and also year of cART initiation); proportion of time with VL ≤ 400 c/mL (with time-updated VL).

cART, combination antiretroviral therapy; EE&T, Eastern Europe and Thailand; HR, hazard ratio; NNRTI, nonnucleoside reverse transcriptase inhibitor; PI, protease inhibitor PY, person years; VL, viral load; W&CE, Western and Central Europe.

In the sensitivity analysis, the model with weight-for-age z-score was broadly similar to the BMI model (S2 Table), but the effect of weight-for-age at ART start was attenuated (>0 adjusted hazard ratio [aHR] 0.57 [0.11–2.88], <−3 aHR 1.07 [0.32–3.63], unknown aHR 1.21 [0.46–3.15] versus −3–0, *p* = 0.875).

### AIDS-defining events

Among those without an AIDS diagnosis by the start of cART (*n* = 2,863), 278 AIDS-defining events were reported in 237 children: 113 (41%) were from Thailand, 75 (27%) in the UK or Ireland, 48 (17%) in Russia or Ukraine, and 42 (15%) in the rest of W&CE. The overall rate of first AIDS-defining event was 1,549 (95% CI 1,364–1,760) per 100,000 person-years and was higher in the first 6 months of cART (7,305 [6,005–8,887]) compared to later (984 [832–1,163], *p* < 0.001). The most commonly reported AIDS events were encephalopathy (38 events in 38 children, 14%), tuberculosis (TB)(34 events in 32 children, 12%), and wasting syndrome (31 events in 30 children, 11%) (Table 5).

**Table 5:** Rates of AIDS-defining events among children with no previous AIDS diagnosis at initiation of cART, *N* = 2,863.

Event	Number of children with an event (number of events)	Rate of first event per 100,000 PY (95% CI)
TOTAL	237 (278)	1,549 (1,364–1,760)
Encephalopathy attributed to HIV	38 (38)	233 (170–321)
*Mycobacterium tuberculosis* of any site, pulmonary (≥6 years), disseminated, or extrapulmonary	32 (34)	195 (138–276)
Wasting syndrome attributed to HIV	30 (31)	184 (128–263)
Bacterial infections, multiple or recurrent (<6 years)	27 (32)	166 (114–241)
PJP	23 (23)	140 (93–211)
Cytomegalovirus disease (other than liver, spleen, or nodes), onset at age >1 month, or retinitis (with loss of vision)	11 (13)	67 (37–121)
Mycobacterium, other species, or unidentified species, disseminated or extrapulmonary	11 (11)	67 (37–121)
Cryptococcosis, extrapulmonary	8 (11)	29 (24–97)
Candidiasis of bronchi, trachea, esophagus, or lungs	6 (6)	36 (16–81)
Cryptosporidiosis, chronic intestinal (>1 month duration)	3 (3)	18 (6–56)
*M*. *avium* complex or *M*. *kansasii*, disseminated or extrapulmonary	3 (3)	18 (6–56)
Progressive multifocal leukoencephalopathy	3 (3)	18 (6–56)
Herpes simplex virus: chronic ulcers (>1 month duration) or bronchitis, pneumonitis, or esophagitis (onset at age >1 month)	2 (2)	12 (3–49)
Kaposi sarcoma	2 (3)	12 (3–48)
Lymphoma, immunoblastic (or equivalent term)	2 (2)	12 (3–48)
Pneumonia, recurrent (≥6 years)	1 (1)	6 (1–43)
*Salmonella spp*. septicemia, recurrent	1 (1)	6 (1–43)
Undefined event	61 (61)	377 (294–485)

cART, combination antiretroviral therapy; PJP, *Pneumocystis jirovecii* pneumonia.

Among the 663 children with an AIDS diagnosis prior to cART initiation, there were a further 80 AIDS-defining events after initiation of cART in 66 (10%) children. The most common events were encephalopathy (21 events in 21 children, 26%), multiple/recurrent bacterial infections (11 events in 10 children, 14%), and TB (11 events in 8 children, 14%); 61 events had a missing cause.

### Risk factors for first AIDS-defining events after cART initiation in those with no previous AIDS diagnosis

In multivariable analysis, factors associated with increased risk of the first AIDS-defining event within 6 months of cART initiation were severe immunosuppression and lower BMI-for-age z-score (<−3 versus 0–−3) at cART initiation (S3 Table). Factors associated with increased risk after 6 months of cART were earlier year of cART initiation, initiating cART on an NNRTI-based regimen (versus any other), higher viral load at cART initiation, severe current immune suppression for age for those in EE&T, current viral load >400 c/mL, and lower current BMI-for-age z-score (S4 Table).

## Discussion

Our study included more than 3,500 children and young adults with HIV infection followed across 16 Western, Central, and Eastern European countries and Thailand. Median age at cART initiation was 5 years and median follow-up 5 years, with a quarter of participants being followed for ≥9 years. The median year of birth was 2000, with a quarter being born before 1997; thus, many children were born before the introduction of cART, and outcomes in older children who survived reflect the first cohort of patients with perinatal HIV progressing to adult life [13]. Nearly half of all deaths (46%) and 42% of first AIDS-defining events were within 6 months of cART start in our study. We found that multivariable predictors of early death were very young age, residence in Eastern European countries and Thailand, AIDS diagnosis at cART initiation, initiating cART on an NNRTI-based regimen, severe immune suppression for age, and low BMI-for-age z-score at cART initiation. Time-updated (current) severe immune suppression for age and low BMI-for-age z-score were also associated with late death, as was current viral load >400 c/mL.

Our results confirm that mortality rates in children starting treatment across the European region and Thailand fell markedly since the introduction of cART in 1996 but also suggest that they have remained stable since 2006, at 122 and 435 deaths per 100,000 person-years, respectively. Our 5-year survival probability after initiating cART was 97.6% overall, and was higher in W&CE countries than EE&T. These findings are consistent with results from other studies. For example, a study of HIV-infected youth in the US reported mortality rates of 660 per 100,000 person years (PY) for the period 2008–2014 and a 5-year survival probability of 97.6% [16], and another US study reported survival probabilities of 76% at 6 years for those born in 1991–1996 and exposed to mono or dual therapy, and 91% at 6 years for those born in 1997–2004 and exposed to cART [3].

Interestingly, our results suggest that mortality rates (and also incidence of first AIDS events) in children initiating cART in the European region and Thailand have not declined since 2006. It is encouraging that the proportion of children presenting late decreased over time, but from 2011 onwards, 1 in 7 children aged <5 years and 1 in 5 children aged ≥5 years at cART initiation still presented with CD4% < 15 or CD4 count <200 cells/mm^3^, despite the CD4 threshold for initiating treatment increasing over time [17]. This highlights the ongoing importance of identifying and testing children most at risk of HIV. Enhanced prophylaxis against infections has been shown to reduce the risk of early death in adults and children with advanced disease in low-income countries [18] and may be relevant in our high-income setting, especially as a third of patients in our study were born abroad, many in sub-Saharan Africa. Further work is needed to ascertain possible contributing factors, such as late HIV diagnosis, suboptimal immune reconstitution despite cART, late access to cART, nonadherence, ART toxicity, and socioeconomic factors. Our results suggest that mortality rates in HIV-infected children initiating cART in the European region are 3–12 times higher than in the general population, as the mortality rate for 0–14-year-olds in 27 countries in the European Union (EU) in 2013 was 35 per 100,000 person-years [19]. Similarly, higher rates of mortality have been found in perinatally HIV-infected older youth compared to the general population in the US [20].

Almost half of all deaths (46%) occurred in the first 6 months of cART, with the majority already having an AIDS diagnosis by the time of cART initiation. Indeed, AIDS may have been an indication for initiating treatment at the time, and with guidelines now recommending universal treatment for HIV [21], it is unlikely that we would observe so many deaths now, with a higher proportion of children being diagnosed earlier (when asymptomatic), through screening. The Therapeutic Research, Education and AIDS Training (TREAT) Asia Pediatric HIV Observational Database (TApHOD) cohort of children in Asia-Pacific found similar results [22], and very young age has been found to predict mortality in other studies [23–26]. In our study, three-quarters of early deaths were due to HIV-related infections, with bacterial/sepsis-related causes being the most common, whilst 22% were due to other HIV-related causes, and only 1 death had a cause not directly related to HIV. Some of these early deaths may have been associated with immune reconstitution inflammatory syndrome [27,28]. The causes of early death contrast with the deaths after 6 months, of which 14% were not directly related to HIV. The trend in causes of death (i.e., HIV-related infections, other HIV-related causes, and not HIV related) did not change over calendar periods, although we had limited power for this. Similarly, we did not detect a decline over the calendar period in the proportion of deaths attributed to AIDS, in contrast to other studies [2,3]. One study of adults with HIV found different trends in the proportion of deaths due to AIDS by region across Europe and Argentina [29], with higher risk of mortality from AIDS-related causes in Eastern Europe and higher non-AIDS mortality in northern Europe.

The strongest predictors of mortality in our analysis were having an AIDS diagnosis at cART initiation, current severe immune suppression for age, and being underweight. These are known risk factors that have been consistently reported in many earlier HIV cohorts [3,20,22]. As expected, children in W&CE countries had a lower risk of death within the first 6 months of cART initiation, perhaps due to better access to medical care, a higher standard of clinical care, higher nutritional status, and better tolerated combinations of cART. One key component of clinical care is vaccination against infectious diseases among children with HIV, and this remains an area in which improvements can be made [30]. Of concern, our findings indicate a raised mortality risk in the first year of life, independent of AIDS diagnosis and immune suppression for age, similar to other study findings [31,32]. This suggests the need for continued vigilance in this group.

Among those who did not have AIDS prior to initiating treatment, 6% of children had a subsequent AIDS-defining event, with the most commonly reported events being encephalopathy, TB, and wasting syndrome. Predictors of first AIDS-defining event were similar to those found for death.

Our study has a number of limitations. We analysed observational data from several cohorts across the European region and Thailand, but events were distributed over a long time period and we lacked statistical power to investigate trends over calendar time of patterns and causes of death in some analyses. Data were not collected in a standardised way across cohorts, but we took time to verify causes of death with reporting clinicians, and each cohort had their own system for data validation and querying. We were unable to analyse data on serious adverse events and CDC disease stage B events, as these were incompletely collected across cohorts. For a small number of children with an AIDS event, information on the type of AIDS event was not available. Around half (52%) of patients came from the UK/Ireland or Thailand, but these countries accounted for 81% of the deaths observed. Conversely, 6 (38%) of the 16 countries that observed no deaths at all (Germany, Greece, the Netherlands, Portugal, Romania, and Sweden) only accounted for 9% of patients.

In conclusion, in our study of more than 3,500 children across the European region and Thailand, mortality rates fell after the introduction of cART in 1997 but have remained stable since 2006, and the prevalence of low CD4 at initiation of cART decreased over the period. Five-year survival probability after initiating cART across the whole period studied was 97.6%. Almost half (46%) of the 94 deaths observed were in the first 6 months of cART, suggesting that close clinical follow-up is recommended over the first 6 months after cART initiation, with enhanced prophylaxis against infections in those presenting with advanced disease. The indication of raised early mortality risk in infants and those in EE&T raises concern and warrants vigilance. It highlights the need to direct additional clinical resources to the care of these groups, as well as further prospective studies evaluating morbidity and mortality in older adolescents.

## Supporting information

S1 STROBE Checklist (DOC)

S1 Text EPPICC Concept Sheet: Mortality and AIDS-defining events in children after ART initiation. ART, antiretroviral therapy; EPPICC, European Pregnancy and Paediatric HIV Cohort Collaboration.
(DOC)

S2 Text Writing group members and collaborating cohorts. (DOCX)

S1 Fig Mortality rates (95% CI) by calendar year of follow-up, 2000–2013, (A) within the first 6 months after cART and (B) after 6 months of cART. cART, combination antiretroviral therapy.
(TIF)

S1 Table Rates and baseline risk factors for death within 6 months of cART initiation, using weight for age instead of BMI for age. cART, combination antiretroviral therapy. (DOCX)

S2 Table Rates and risk factors (baseline and time updated) for death after 6 months of cART, using weight for age instead of BMI for age. cART, combination antiretroviral therapy. (DOCX)

S3 Table Rates and baseline risk factors for first AIDS-defining event within 6 months after cART initiation. cART, combination antiretroviral therapy. (DOCX)

S4 Table Rates and risk factors (baseline and time updated) for first AIDS-defining event after 6 months of cART. cART, combination antiretroviral therapy. (DOCX)

## Abbreviations

aHR: adjusted hazard ratio
ART: antiretroviral therapy
cART: combination antiretroviral therapy
CDC: US Centers for Disease Control
CD4%: CD4 lymphocyte percentage
CMV: cytomegalovirus
EE&T: Eastern Europe and Thailand
EPPICC: European Pregnancy and Paediatric HIV Cohort Collaboration
EU: European Union
HR: hazard ratio
NNRTI: nonnucleoside reverse transcriptase inhibitor
PJP: *Pneumocystis jirovecii* pneumonia
PY: person years
TB: tuberculosis
VL: viral load
W&CE: Western and Central Europe
WHO: World Health Organization
