# Supplementary material for: Long-term trends in mortality and AIDS-defining events after combination ART initiation among children and adolescents with perinatal HIV infection in 17 middle- and high-income countries in Europe and Thailand: A cohort study
Source: PLoS Med. 2018 Jan 30;15(1):e1002491. doi: 10.1371/journal.pmed.1002491 (PMC5790238; doi:10.1371/journal.pmed.1002491)
Supplement: S1 Text — ART, antiretroviral therapy; EPPICC, European Pregnancy and Paediatric HIV Cohort Collaboration. (DOC) [file pmed.1002491.s002.doc]

**S1 Text: European Pregnancy and Paediatric HIV Cohort Collaboration (EPPICC) Concept Sheet: Mortality and AIDS-defining events in children after ART initiation**

| 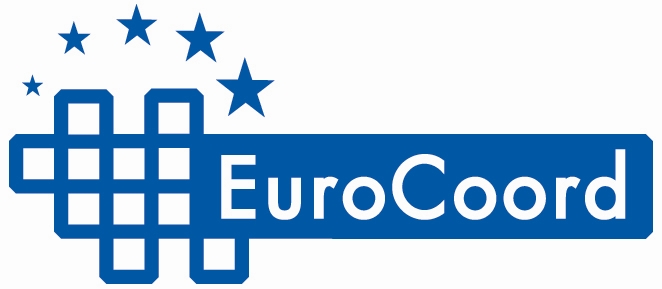 | European Pregnancy and Paediatric HIV Cohort Collaboration (EPPICC) **Proposal for new projects** | 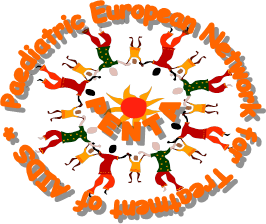 |
| --- | --- | --- |

| Proposal title | **Mortality and AIDS-defining events in children after ART initiation** |
| --- | --- |
| Submitted by (date) | Ali Judd (1 April 2013) |
| Cohort affiliation, if applicable | EPPICC |
| Members of proposed project team, and roles | Project lead: Ali  Project statistician: Lizzie and Ruth  Representatives from cohorts participating in the project. |
| Background, including any preliminary work undertaken | In studies of adults receiving ART, current CD4 count has been consistently shown to be the strongest prognostic factor of mortality and progression to AIDS. Furthermore, patients on treatment long-term with CD4 count above 500 cells/mm^3^ were found to have similar mortality rates as the general population .  However, there are limited data in children on long-term survival after ART start, as well as the prognostic effects of time-dependent covariates. In a study of nearly 600 children from the Programs for HIV Prevention &Treatment (PHPT) in Thailand, the estimated 3-years mortality probability was 27% for those starting treatment aged <12 months compared to 5% at age >1 year . Another Thai cohort including 1752 children estimated an overall 5-year mortality rate of 8.3%, with more than a third of deaths occurring within the first 3 months of treatment . The mortality rate in this study increased substantially when current CD4 percent fell below 5% with little trend at higher CD4 values, though the confidence intervals were very wide due to the small number of deaths.  Furthermore, while numerous studies have described the spectrum of AIDS-defining illnesses in children pre-ART, clinical progression during treatment has not been well documented. The risk of developing AIDS/death at 1 year was estimated at 1.6% among 124 infants in European cohorts who started treatment early before age 3 months .  Improved understanding of long-term disease progression in children on ART treatment and key prognostic factors are important for informing clinical management guidelines. |
| Key aims/research questions | 1) For different types of AIDS-defining illness (for example, malignancy), to describe the frequency and rate of events after starting ART.  2) To describe over time from ART initiation  - overall risk of all-cause mortality and causes of death  - overall risk of progression to initial AIDS diagnosis among children who were AIDS-free at start of ART  - overall risk of developing a clinically new AIDS event.  3) To identify prognostic factors for progression to death and to AIDS/death, considering both baseline and time-dependent covariates (for example, current CD4 count/percent, viral load, weight-for-age z-score). |
| Justification for use of EPPICC data | The large sample size in EPPICC is required to provide sufficient number of deaths and AIDS events to assess long-term disease progression; in the UK/Irish Collaborative HIV Paediatric Study (CHIPS), there were only 25 deaths among children starting ART naïve from 1996 onwards, 12 occurring after 6 months of treatment initiation. |
| Study population, including estimated sample size if possible | Children and adolescents aged <18 years starting ART naïve from 1996 onwards with ≥3 drugs simultaneously (excluding with unboosted PI-based regimens), with at least 1 day of follow-up after ART start.  Estimated sample size 2500-3000 across EPPICC cohorts (extrapolating from sample size of ~1100 in CHIPS). |
| Data required | Baseline demographic and clinical characteristics; ART history; CD4, viral load, height and weight measurements; AIDS-defining events; death and cause of death; current follow-up status and date of last clinic visit.  Data will be provided in HICDEP format. |
| Resources required  (eg statistical support) | None |
| Deliverables and timelines | Analysis to be started in 2015. Paper to be drafted end of 2017. |
| Details on how this study will be funded | Eurocoord |
| References | 1. Lewden C, Chene G, Morlat P, Raffi F, Dupon M, Dellamonica P*, et al.* HIV-infected adults with a CD4 cell count greater than 500 cells/mm3 on long-term combination antiretroviral therapy reach same mortality rates as the general population. *J Acquir Immune Defic Syndr* 2007,**46**:72-77.  2. Collins IJ, Jourdain G, Hansudewechakul R, Kanjanavanit S, Hongsiriwon S, Ngampiyasakul C*, et al.* Long-term survival of HIV-infected children receiving antiretroviral therapy in Thailand: a 5-year observational cohort study. *Clin Infect Dis* 2010,**51**:1449-1457.  3. Lumbiganon P, Kariminia A, Aurpibul L, Hansudewechakul R, Puthanakit T, Kurniati N*, et al.* Survival of HIV-infected children: a cohort study from the Asia-Pacific region. *J Acquir Immune Defic Syndr* 2011,**56**:365-371.  4. Goetghebuer T, Haelterman E, Le Chenadec J, Dollfus C, Gibb D, Judd A*, et al.* Effect of early antiretroviral therapy on the risk of AIDS/death in HIV-infected infants. *AIDS* 2009,**23**:597-604. |
