# Supplementary material for: Long-term trends in mortality and AIDS-defining events after combination ART initiation among children and adolescents with perinatal HIV infection in 17 middle- and high-income countries in Europe and Thailand: A cohort study
Source: PLoS Med. 2018 Jan 30;15(1):e1002491. doi: 10.1371/journal.pmed.1002491 (PMC5790238; doi:10.1371/journal.pmed.1002491)
Supplement: S2 Text — (DOCX) [file pmed.1002491.s003.docx]

**S2 Text: Writing Group members and collaborating cohorts**

Writing Group (consisting of Project Team first (ordered alphabetically by name except for the first and last author), and also other Writing Group members (ordered alphabetically by cohort name):

*Project Team:* Ali Judd (co-lead of EPPICC), Elizabeth Chappell (EPPICC statistician), Anna Turkova (PENTA), Sophie Le Coeur (Thailand Program for HIV Prevention and Treatment (PHPT), Thailand), Antoni Noguera-Julian (CoRISPE-cat cohort, Spain), Tessa Goetghebuer (Hospital St Pierre paediatric cohort, Belgium), Katja Doerholt (Collaborative HIV Paediatric Study (CHIPS), UK and Ireland), Luisa Galli (Italian Register for HIV Infection in Children, Italy), Ruth Goodall (EPPICC senior statistician).

*Other Writing Group members:* Dasja Pajkrt (ATHENA paediatric cohort, Netherlands); Laura Marques (Centro Hospitalar do Porto, Portugal); Intira J. Collins, Diana M. Gibb (Collaborative HIV Paediatric Study (CHIPS) and National Study of HIV in Pregnancy and Childhood (NSHPC), UK & Ireland); Maria Isabel González Tome, Marisa Navarro (CoRISPE-1, rest of Spain cohort, Spain); Josiane Warszawski (French Perinatal Cohort Study, France); Christoph Königs (German Pediatric and Adolescent HIV cohort, Germany); Vana Spoulou (Greece Cohort, Greece); Filipa Prata (Hospital de Santa Maria/CHLN, Lisbon, Portugal); Elena Chiappini (Italian Register for HIV infection in children, Italy); Lars Naver (Karolinska University Hospital, Stockholm, Sweden); Carlo Giaquinto, Claire Thorne (Paediatric European Network for the Treatment of AIDS (PENTA), Italy); Magdalena Marczynska (Polish paediatric cohort, Poland); Liubov Okhonskaia (Republican Hospital of Infectious Diseases, St Petersburg, Russia); Klara Posfay-Barbe (Swiss Mother and Child HIV Cohort Study, Switzerland); Pradthana Ounchanum, Pornchai Techakunakorn (Thailand Program for HIV Prevention and Treatment (PHPT) Study Group, Thailand); Galina Kiseleva, Ruslan Malyuta, Alla Volokha (Ukraine Paediatric HIV Cohort Study, Odessa, Ukraine); Luminita Ene (“Victor Babes” Hospital Cohort, Romania).

**COLLABORATING COHORTS**

**Belgium:** Hospital St Pierre Cohort, Brussels: Tessa Goetghebuer, MD, PhD; Marc Hainaut, MD PhD; Evelyne Van der Kelen, Research nurse; Marc Delforge, data manager.

**France:** French Perinatal Cohort Study/Enquête Périnatale Française, ANRS EPF-CO10. Coordinating center, INSERM U1018, team 4 : Josiane Warszawski, Jerome Le Chenadec, Elisa Ramos, Olivia Dialla, Thierry Wack, Corine Laurent, Lamya Ait si Selmi, Isabelle Leymarie, Fazia Ait Benali, Maud Brossard, Leila Boufassa

Participating sites (hospital name, city, main investigator): Hôpital Louis Mourier, Colombes, Dr Corinne Floch-Tudal; Groupe Hospitalier Cochin Tarnier Port-Royal, PARIS, Dr Ghislaine Firtion; Centre Hospitalier Intercommunal, Creteil, Dr Isabelle Hau; Centre Hospitalier Général, Villeneuve Saint Georges, Dr Anne Chace; Centre Hospitalier Général- Hôpital Delafontaine, Saint-Denis, Dr Pascal Bolot; Groupe Hospitalier Necker, Paris, Pr Stéphane Blanche; Centre hospitalier Francilien Sud, Corbeil Essonne, Dr Michèle Granier; Hôpital Antoine Béclère, Clamart, Pr Philippe Labrune; Hôpital Jean Verdier, Bondy, Dr Eric Lachassine; Hôpital Trousseau, Paris, Dr Catherine Dollfus; Hôpital Robert Debré, Paris, Dr Martine Levine; Hôpital Bicêtre, Le Kremlin Bicëtre, Dr Corinne Fourcade; Centre Hospitalier Intercommunal, Montreuil, Dr Brigitte Heller- Roussin; Centre Hospitalier Pellegrin, Bordeaux, Dr Camille Runel-Belliard; CHU Paule de Viguier, Toulouse, Dr Joëlle Tricoire; CHU Hôpital de l'Archet II, Nice, Dr Fabrice Monpoux; Groupe Hospitalier de la Timone, Marseille; CHU Hôpital Jean Minjoz, Besancon, Dr Catherine Chirouze; CHU Nantes Hotel Dieu, Nantes, Dr Véronique Reliquet; CHU Caen, Caen, Pr Jacques Brouard; Institut d’Hématologie et Oncologie Pédiatrique, Lyon, Dr Kamila Kebaili; CHU Angers, Angers, Dr Pascale Fialaire; CHR Arnaud de Villeneuve, Montpellier, Dr Muriel Lalande; CHR Jeanne de Flandres, Lille, Dr Françoise Mazingue; Hôpital Civil, Strasbourg, Dr Maria Luisa Partisani

**Germany:** German Paediatric & Adolescent HIV Cohort (GEPIC): Dr Christoph Königs, Dr Stephan Schultze-Strasser. German clinical centers: Hannover Medical School, Dr. U. Baumann; Pediatric Hospital Krefeld, Dr. T. Niehues; University Hospital Düsseldorf, Dr. J. Neubert; University Hospital Hamburg, Dr. R. Kobbe; Charite Berlin, Dr. C. Feiterna-Sperling; University Hospital Frankfurt, Dr. C. Königs; University Hospital Mannheim, Dr. B. Buchholz; Munich University Hospital, Dr. G. Notheis

**Greece:** Greek cohort: Vana Spoulou.

**Italy:** Italian Register for HIV infection in Children. Coordinators: Maurizio de Martino (Florence), Pier Angelo Tovo (Turin). Participants: Osimani Patrizia (Ancona), Domenico Larovere (Bari), Maurizio Ruggeri (Bergamo), Giacomo Faldella, Francesco Baldi (Bologna) Raffaele Badolato (Brescia), Carlotta Montagnani, Elisabetta Venturini, Catiuscia Lisi (Florence), Antonio Di Biagio, Lucia Taramasso (Genua), Vania Giacomet, Paola Erba, Susanna Esposito, Rita Lipreri, Filippo Salvini, Claudia Tagliabue (Milan), Monica Cellini (Modena), Eugenia Bruzzese, Andrea Lo Vecchio (Naples), Osvalda Rampon, Daniele Donà (Padua), Amelia Romano (Palermo), Icilio Dodi (Parma), Anna Maccabruni (Pavia), Rita Consolini (Pisa), Stefania Bernardi, Hyppolite Tchidjou Kuekou, Orazio Genovese (Rome), Paolina Olmeo (Sassari), Letizia Cristiano (Taranto), Antonio Mazza (Trento), Clara Gabiano, Silvia Garazzino (Turin), Antonio Pellegatta (Varese)

**Netherlands:** The ATHENA database is maintained by Stichting HIV Monitoring and supported by a grant from the Dutch Ministry of Health, Welfare and Sport through the Centre for Infectious Disease Control of the National Institute for Public Health and the Environment.

**CLINICAL CENTRES (PAEDIATRIC CARE)**

**Emma Kinderziekenhuis, Academic Medical Centre of the University of Amsterdam:** *HIV treating physicians:* D. Pajkrt, H.J. Scherpbier. *HIV nurse consultants:* A.M. Weijsenfeld, A. van der Plas. *HIV clinical virologists/chemists:* S. Jurriaans, N.K.T. Back, H.L. Zaaijer, B. Berkhout, M.T.E. Cornelissen, C.J. Schinkel, K.C.wolthers. **Erasmus MC–Sophia, Rotterdam:** *HIV treating physicians:* P.L.A. Fraaij, A.M.C. van Rossum. *HIV nurse consultants:* L.C. van der Knaap, E.G. Visser. *HIV clinical virologists/chemists:* C.A.B. Boucher, M.P.G Koopmans, J.J.A van Kampen, S.D. Pas. **Radboudumc, Nijmegen:** *HIV treating physicians:* S.S.V. Henriet, M. van de Flier, K. van Aerde. *HIV nurse consultants:* R. Strik-Albers. *HIV clinical virologists/chemists:* J. Rahamat-Langendoen, F.F. Stelma. **Universitair Medisch Centrum Groningen, Groningen:** *HIV treating physicians: E.* H. Schölvinck. *HIV nurse consultants:* H. de Groot-de Jonge. *HIV clinical virologists/chemists:* H.G.M. Niesters, C.C. van Leer-Buter, M. Knoester. **Wilhelmina Kinderziekenhuis, UMCU, Utrecht:** *HIV treating physicians:* L.J. Bont, S.P.M. Geelen, T.F.W. Wolfs. *HIV nurse consultants:* N. Nauta. *HIV clinical virologists/chemists:* C.W. Ang, R. van Houdt, A.M. Pettersson, C.M.J.E. Vandenbroucke-Grauls.

**COORDINATING CENTRE**

*Director:* P. Reiss. *Data analysis:* D.O. Bezemer, A.I. van Sighem, C. Smit, F.W.M.N. Wit, T.S. Boender. *Data management and quality control:* S. Zaheri, M. Hillebregt, A. de Jong. *Data monitoring:* D. Bergsma, S. Grivell, A. Jansen, M. Raethke, R. Meijering. *Data collection:* L. de Groot, M. van den Akker, Y. Bakker, E. Claessen, A. El Berkaoui, J. Koops, E. Kruijne, C. Lodewijk, L. Munjishvili, B. Peeck, C. Ree, R. Regtop, Y. Ruijs, T. Rutkens, M. Schoorl, A. Timmerman, E. Tuijn, L. Veenenberg, S. van der Vliet, A. Wisse, T. Woudstra. *Patient registration:* B. Tuk.

**Poland:** Polish paediatric cohort: Head of the team: Prof Magdalena Marczyńska, MD, PhD.  Members of the team: Jolanta Popielska, MD, PhD; Maria Pokorska-Śpiewak, MD, PhD; Agnieszka Ołdakowska, MD, PhD; Konrad Zawadka, MD, PhD; Urszula Coupland, MD, PhD.  Administration assistant: Małgorzata Doroba.  Affiliation: Medical University of Warsaw, Poland, Department of Children’s Infectious Diseases; Hospital of Infectious Diseases in Warsaw, Poland.

**Portugal:** Centro Hospitalar do Porto:Laura Marques, Carla Teixeira, Alexandre Fernandes.

**Portugal:** Hospital de Santa Maria/CHLN: Filipa Prata.

**Romania:** "Victor Babes" Hospital Cohort, Bucharest: Dr Luminita Ene.

**Russia:** Federal State-owned Institution "Republican Clinical Infectious Diseases Hospital" of the Ministry of Health of the Russian Federation, St Petersburg: Liubov Okhonskaia, Evgeny Voronin, Milana Miloenko, Svetlana Labutina

**Spain:** CoRISPE-cat, Catalonia: financial support for CoRISPE-cat was provided by the Instituto de Salud Carlos III through the Red Temática de Investigación Cooperativa en Sida. Members: Hospital Universitari Vall d’Hebron, Barcelona (Pere Soler-Palacín, Maria Antoinette Frick and Santiago Pérez-Hoyos (statistician)), Hospital Universitari del Mar, Barcelona (Antonio Mur, Núria López), Hospital Universitari Germans Trias i Pujol, Badalona (María Méndez), Hospital Universitari JosepTrueta, Girona (Lluís Mayol), Hospital Universitari Arnau de Vilanova, Lleida (Teresa Vallmanya), Hospital Universitari Joan XXIII, Tarragona (Olga Calavia), Consorci Sanitari del Maresme, Mataró (Lourdes García), Hospital General de Granollers (Maite Coll), Corporació Sanitària Parc Taulí, Sabadell (Valentí Pineda), Hospital Universitari Sant Joan, Reus (Neus Rius), Fundació Althaia, Manresa (Núria Rovira), Hospital Son Espases, Mallorca (Joaquín Dueñas) and Hospital Sant Joan de Déu, Esplugues (Clàudia Fortuny, Antoni Noguera-Julian).

**Spain:** CoRISPE-S and Madrid cohort: María José Mellado, Luis Escosa, Milagros García Hortelano, Talía Sainz (Hospital La Paz);María Isabel González- Tomé, Pablo Rojo, Daniel Blázquez (Hospital Doce de Octubre, Madrid); José Tomás Ramos (Hospital Clínico San Carlos, Madrid); Luis Prieto, Sara Guillén (Hospital de Getafe); María Luisa Navarro, Jesús Saavedra, Mar Santos, Mª Angeles Muñoz, Beatriz Ruiz, Carolina Fernandez Mc Phee, Santiago Jimenez de Ory,Susana Alvarez (Hospital Gregorio Marañón); Miguel Ángel Roa (Hospital de Móstoles); José Beceiro (Hospital Príncipe de Asturias, Alcalá de Henares); Jorge Martínez (Hospital Niño Jesús, Madrid); Katie Badillo (Hospital de Torrejón); Miren Apilanez (Hospital de Donostia, San Sebastián); Itziar Pocheville (Hospital de Cruces, Bilbao); Elisa Garrote (Hospital de Basurto, Bilbao); Elena Colino (Hospital Insular Materno Infantil, Las Palmas de Gran Canaria); Jorge Gómez Sirvent (Hospital Virgen de la Candelaria, Santa Cruz de Tenerife); Mónica Garzón, Vicente Román (Hospital de Lanzarote); Abián Montesdeoca, Mercedes Mateo (Complejo Universitario de Canarias, La Laguna-Tenerife),María José Muñoz, Raquel Angulo (Hospital de Poniente, El Ejido); Olaf Neth, Lola Falcón (Hospital Virgen del Rocio, Sevilla); Pedro Terol (Hospital Virgen de la Macarena, Sevilla); Juan Luis Santos (Hospital Virgen de las Nieves, Granada); David Moreno (Hospital Carlos Haya, Málaga); Francisco Lendínez (Hospital de Torrecárdenas, Almería); Ana Grande (Complejo Hospitalario Universitario Infanta Cristina, Badajoz); Francisco José Romero (Complejo Hospitalario de Cáceres); Carlos Pérez (Hospital de Cabueñes, Gijón); Miguel Lillo (Hospital de Albacete); Begoña Losada (Hospital Virgen de la Salud, Toledo); Mercedes Herranz (Hospital Virgen del Camino, Pamplona); Matilde Bustillo, Carmelo Guerrero (Hospital Miguel Servet, Zaragoza); Pilar Collado (Hospital Clínico Lozano Blesa, Zaragoza); José Antonio Couceiro (Complejo Hospitalario de Pontevedra); Amparo Pérez, Ana Isabel Piqueras, Rafael Bretón, Inmaculada Segarra (Hospital La Fe, Valencia); César Gavilán (Hospital San Juan de Alicante); Enrique Jareño (Hospital Clínico de Valencia); Elena Montesinos (Hospital General de Valencia); Marta Dapena (Hospital de Castellón); Cristina Álvarez (Hospital Marqués de Valdecilla, Santander); Ana Gloria Andrés (Hospital de León); Víctor Marugán, Carlos Ochoa (Hospital de Zamora); Santiago Alfayate, Ana Isabel Menasalvas (Hospital Virgen de la Arrixaca, Murcia); Elisa de Miguel (Complejo Hospitalario San Millán-San Pedro, Logroño) and Paediatric HIV-BioBank integrated in the Spanish AIDS Research Network and collaborating Centers.

**Funding:** This work has been partially funded by the Fundación para la Investigación y Prevención de SIDA en España (FIPSE) (FIPSE 3608229/09 , FIPSE 240800/09, FIPSE 361910/10), Red Temática de Investigación en SIDA (RED RIS) supported by Instituto de Salud Carlos III (ISCIII) (RD12/0017/0035 and RD12/0017/0037), project as part of the Plan R+D+I and cofinanced by ISCIII- Subdirección General de Evaluación and Fondo Europeo de Desarrollo Regional (FEDER),Mutua Madrileña 2012/0077, Gilead Fellowship 2013/0071, FIS PI15/00694 ,CoRISpe (RED RIS RD06/0006/0035 y RD06/0006/0021).

**Sweden:** Karolinska University Hospital, Stockholm (Lars Naver, Sandra Soeria-Atmadja, Vendela Hagås).

**Switzerland:** *Members of the Swiss HIV Cohort Study (SHCS) and the Swiss Mother and Child HIV Cohort Study:* Aebi-Popp K, Asner S, Aubert V, Battegay M, Baumann M, Bernasconi E, Böni J, Brazzola P, Bucher HC, Calmy A, Cavassini M, Ciuffi A, Duppenthaler A, Dollenmaier G, Egger M, Elzi L, Fehr J, Fellay J, Francini K, Furrer H, Fux CA, Grawe C, Günthard HF (President of the SHCS), Haerry D (deputy of "Positive Council"), Hasse B, Hirsch HH, Hoffmann M, Hösli I, Kahlert C, Kaiser L, Keiser O, Klimkait T, Kovari H, Kouyos RD, Ledergerber B, Martinetti G, Martinez de Tejada B, Metzner KJ, Müller, Nicca D, Paioni P, Pantaleo G, Polli Ch, Posfay-Barbe K, Rauch A, Rudin C (Chairman of the Mother & Child Substudy), Schmid P, Scherrer AU (Head of Data Centre), Speck R, Tarr P, Thanh Lecompte M, Trkola A, Vernazza P, Wagner N, Wandeler G, Weber R, Wyler CA, Yerly S. *Funding:* the Swiss HIV Cohort Study is supported by the Swiss National Science Foundation (grant #148522), and by the SHCS research foundation.

**Thailand:** Program for HIV Prevention & Treatment (PHPT). Participating hospitals: Lamphun: Pornpun Wannarit; Phayao Provincial Hospital: Pornchai Techakunakorn; Chiangrai Prachanukroh: Rawiwan Hansudewechakul; Chiang Kham: Vanichaya Wanchaitanawong; Phan: Sookchai Theansavettrakul; Mae Sai: Sirisak Nanta; Prapokklao: Chaiwat Ngampiyaskul; Banglamung: Siriluk Phanomcheong; Chonburi: Suchat Hongsiriwon; Rayong: Warit Karnchanamayul; Bhuddasothorn Chacheongsao: Ratchanee Kwanchaipanich; Nakornping: Suparat Kanjanavanit; Somdej Prapinklao: Nareerat Kamonpakorn, Maneeratn Nantarukchaikul; Bhumibol Adulyadej: Prapaisri Layangool, Jutarat Mekmullica; Pranangklao: Paiboon Lucksanapisitkul, Sudarat Watanayothin; Buddhachinaraj: Narong Lertpienthum; Hat Yai: Boonyarat Warachit; Regional Health Promotion Center 6, Khon Kaen: Sansanee Hanpinitsak; Nong Khai: Sathit Potchalongsin; Samutsakhon: Pimpraphai Thanasiri, Sawitree Krikajornkitti; Phaholpolphayuhasena: Pornsawan Attavinijtrakarn; Kalasin: Sakulrat Srirojana; Nakhonpathom: Suthunya Bunjongpak; Samutprakarn: Achara Puangsombat; Mahasarakam: Sathaporn Na-Rajsima; Roi-et: Pornchai Ananpatharachai; Sanpatong: Noppadon Akarathum; Vachira Phuket: Weerasak Lawtongkum; Chiangdao: Prapawan Kheunjan, Thitiporn Suriyaboon, Airada Saipanya.

Data management team: Kanchana Than-in-at, Nirattiya Jaisieng, Rapeepan Suaysod, Sanuphong Chailoet, Naritsara Naratee, and Suttipong Kawilapat.

**Ukraine:** Paediatric HIV Cohort: Dr T. Kaleeva, Dr Y. Baryshnikova (Odessa Regional Centre for HIV/AIDS, Dr S. Soloha (Donetsk Regional Centre for HIV/AIDS), Dr N. Bashkatova (Mariupol AIDS Center), Dr I. Raus (Kiev City Centre for HIV/AIDS), Dr O. Glutshenko, Dr Z. Ruban (Mykolaiv Regional Centre for HIV/AIDS), Dr N. Prymak (Kryvyi Rih), Dr G. Kiseleva (Simferopol), Dr H. Bailey (UCL, London, UK). Funding acknowledgement: PENTA Foundation.

**UK & Ireland:** Collaborative HIV Paediatric Study (CHIPS): CHIPS is funded by the NHS (London Specialised Commissioning Group) and has received additional support from Bristol-Myers Squibb, Boehringer Ingelheim, GlaxoSmithKline, Roche, Abbott, and Gilead Sciences. The MRC Clinical Trials Unit at UCL is supported by the Medical Research Council (<https://www.mrc.ac.uk>) programme number MC_UU_12023/26.

CHIPS Steering Committee: Hermione Lyall, Karina Butler, Katja Doerholt, Caroline Foster, Nigel Klein, Esse Menson, Andrew Riordan, Delane Shingadia, Gareth Tudor-Williams, Pat Tookey, Steve Welch. MRC Clinical Trials Unit: Intira Jeannie Collins, Claire Cook, Donna Dobson, Keith Fairbrother, Diana M. Gibb, Ali Judd, Lynda Harper, Francesca Parrott, Anna Tostevin, Nadine Van Looy.

Participating hospitals: Republic of Ireland: Our Lady's Children’s Hospital Crumlin, Dublin: K Butler, A Walsh. UK: Birmingham Heartlands Hospital, Birmingham: S Scott, Y Vaughan, S Welch; Blackpool Victoria Hospital, Blackpool: N Laycock; Bristol Royal Hospital for Children, Bristol: J Bernatoniene, A Finn, L Hutchison; Calderdale Royal Hospital, Halifax: G Sharpe; Central Middlesex Hospital, London: A Williams; Chelsea and Westminster Hospital, London: EGH Lyall, P Seery; Coventry & Warwickshire University Hospital, Coventry: P Lewis, K Miles; Derbyshire Children’s Hospital, Derby: B Subramaniam; Derriford Hospital, Plymouth: L Hutchinson, P Ward; Ealing Hospital, Middlesex: K Sloper; Eastbourne District General Hospital, Eastbourne: G Gopal; Glasgow Royal Hospital for Sick Children, Glasgow: C Doherty, R Hague, V Price; Great Ormond St Hospital for Children, London: A Bamford, H Bundy, M Clapson, J Flynn, DM Gibb, N Klein, V Novelli, D Shingadia; Halliwell Children’s Centre, Bolton: P Ainsley-Walker; Harrogate District Hospital, Harrogate: P Tovey; Homerton University Hospital, London: D Gurtin; Huddersfield Royal Infirmary, Huddersfield: JP Garside; James Cook Hospital, Middlesbrough: A Fall; John Radcliffe Hospital, Oxford: D Porter, S Segal; King's College Hospital, London: C Ball, S Hawkins; Leeds General Infirmary, Leeds: P Chetcuti, M Dowie; Leicester Royal Infirmary, Leicester: S Bandi, A McCabe; Luton and Dunstable Hospital, Luton: M Eisenhut; Mayday University Hospital, Croydon: J Handforth; Milton Keynes General Hospital, Milton Keynes: PK Roy; Newcastle General Hospital, Newcastle: T Flood, A Pickering; Newham General Hospital, London: S Liebeschuetz; Norfolk & Norwich Hospital, Norwich: C Kavanagh; North Manchester General Hospital, Manchester: C Murphy, K Rowson, T Tan; North Middlesex Hospital, London: J Daniels, Y Lees; Northampton General Hospital, Northampton: E Kerr, F Thompson; Northwick Park Hospital Middlesex; M Le Provost, A Williams; Nottingham City Hospital, Nottingham: L Cliffe, A Smyth, S Stafford; Queen Alexandra Hospital, Portsmouth: A Freeman; Raigmore Hospital, Inverness: T Reddy; Royal Alexandra Hospital, Brighton: K Fidler; Royal Belfast Hospital for Sick Children, Belfast: S Christie; Royal Berkshire Hospital, Reading: A Gordon; Royal Children’s Hospital, Aberdeen: D Rogahn; Royal Cornwall Hospital, Truro: S Harris, L Hutchinson; Royal Devon and Exeter Hospital, Exeter: A Collinson, L Hutchinson; Royal Edinburgh Hospital for Sick Children, Edinburgh: L Jones, B Offerman; Royal Free Hospital, London: V Van Someren; Royal Liverpool Children’s Hospital, Liverpool: C Benson, A Riordan; Royal London Hospital, London: A Riddell; Royal Preston Hospital, Preston: R O’Connor; Salisbury District General Hospital, Salisbury: N Brown; Sheffield Children's Hospital, Sheffield: L Ibberson, F Shackley; Southampton General Hospital, Southampton: SN Faust, J Hancock; St George's Hospital, London: K Doerholt, S Donaghy, K Prime, M Sharland, S Storey; St Luke’s Hospital, Bradford: S Gorman; St Mary’s Hospital, London: EGH Lyall, C Monrose, P Seery, G Tudor-Williams, S Walters; St Thomas’ Hospital (Evelina Children’s Hospital), London: R Cross, E Menson; Torbay Hospital, Torquay: J Broomhall, L Hutchinson; University Hospital Lewisham, London: D Scott, J Stroobant; University Hospital of North Staffordshire, Stoke On Trent: A Bridgwood, P McMaster; University Hospital of Wales, Cardiff: J Evans, T Gardiner; Wexham Park, Slough: R Jones; Whipps Cross Hospital, London: K Gardiner.
