# Supplementary figures and images for: Long-term trends in mortality and AIDS-defining events after combination ART initiation among children and adolescents with perinatal HIV infection in 17 middle- and high-income countries in Europe and Thailand: A cohort study
Source: PLoS Med. 2018 Jan 30;15(1):e1002491. doi: 10.1371/journal.pmed.1002491 (PMC5790238; doi:10.1371/journal.pmed.1002491)

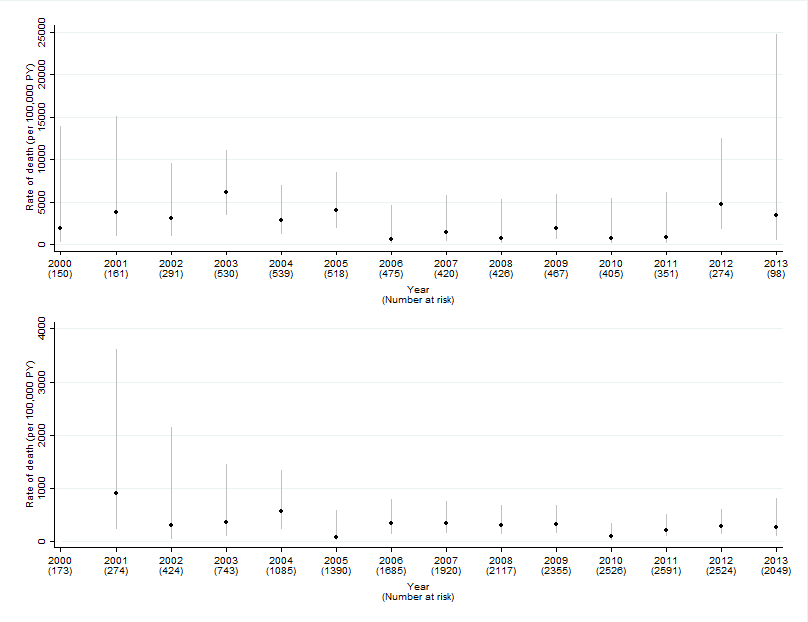

Supplement: S1 Fig — Mortality rates (95% CI) by calendar year of follow-up, 2000–2013, (A) within the first 6 months after cART and (B) after 6 months of cART. cART, combination antiretroviral therapy. (TIF) [file pmed.1002491.s004.tif]
