# Supplementary material for: Long-term trends in mortality and AIDS-defining events after combination ART initiation among children and adolescents with perinatal HIV infection in 17 middle- and high-income countries in Europe and Thailand: A cohort study
Source: PLoS Med. 2018 Jan 30;15(1):e1002491. doi: 10.1371/journal.pmed.1002491 (PMC5790238; doi:10.1371/journal.pmed.1002491)
Supplement: S1 Table — (DOCX) [file pmed.1002491.s005.docx]

S1 Table: Rates and baseline risk factors for death within 6 months of cART initiation, using weight-for-age instead of BMI-for-age

|  | | **N deaths** | **Rate per 100,000 PY (95% CI)** | **Univariable** | | | **Multivariable** | | | |
| --- | --- | --- | --- | --- | --- | --- | --- | --- | --- | --- |
|  |  |  |  | **HR** | **95% CI** | **p** | **HR** | **95% CI** | | **p** |
| **Baseline characteristics at initiation of cART** | | | | | | | | | | |
| Sex | Male | 18 | 2208 (1391-3504) | 0.80 | 0.44-1.47 | 0.469 | 0.81 | | 0.42-1.54 | 0.515 |
|  | Female | 25 | 2769 (1871-4098) | 1.00 | - |  | 1.00 | | - |  |
| Age (per year increase) | <2 years | 16 | 3143 (1925-5130) | 0.43 | 0.25-0.73 | 0.024 | 0.28 | | 0.12-0.62 | 0.018 |
|  | ≥2-<8 years | 12 | 1852 (1052-3261) | 1.27 | 1.04-1.54 |  | 1.26 | | 1.00-1.59 |  |
|  | ≥8 years | 15 | 2674 (1612-4435) | 0.93 | 0.76-1.13 |  | 0.95 | | 0.74-1.22 |  |
| Year of birth (per year increase) | <2003 | 31 | 2777 (1953-3949) | 0.93 | 0.87-1.00 | 0.012 | - | | | |
|  | ≥2003-<2007 | 4 | 1208 (453-3219) | 0.87 | 0.64-1.19 |  |  |  |  |  |
|  | ≥2007 | 8 | 2954 (1477-5906) | 1.55 | 1.12-2.14 |  |  |  |  |  |
| Place of birth | Within country | 27 | 2435 (1670-3551) | 1.00 | - | <0.001 | - | | | |
|  | Abroad | 8 | 1507 (754-3013) | 0.62 | 0.28-1.37 |  |  |  |  |  |
|  | Unknown | 8 | 10172 (5087-20340) | 4.19 | 1.90-9.25 |  |  |  |  |  |
| Ethnicity | Black African | 10 | 1732 (932-3220) | 0.24 | 0.12-0.50 | <0.001 | - | | | |
|  | Asian | 25 | 7215 (4875-10677) | 1.00 | - |  |  |  |  |  |
|  | Other | 6 | 1258 (565-2800) | 0.17 | 0.07-0.43 |  |  |  |  |  |
|  | Unknown | 2 | 630 (158-2520) | 0.09 | 0.02-0.37 |  |  |  |  |  |
| Country group | W&CE | 14 | 1304 (772-2201) | 0.29 | 0.15-0.55 | <0.001 | 0.34 | | 0.15-0.78 | 0.011 |
|  | EE&T | 29 | 4501 (3128-6477) | 1.00 | - |  | 1.00 | | - |  |
| AIDS diagnosis | No AIDS | 17 | 1214 (755-1953) | 1.00 | - | <0.001 | 1.00 | | - | <0.001 |
|  | AIDS | 26 | 8183 (5571-12018) | 6.73 | 3.65-12.43 |  | 4.04 | | 2.11-7.73 |  |
| Year of cART initiation (per year increase) | | - | - | 0.65 | 0.43-0.96 | 0.032 | 0.90 | | 0.57-1.40 | 0.635 |
| Initial regimen | NNRTI-based | 36 | 3312 (2389-4592) | 1.00 | - | 0.008 | 1.00 | | - | 0.092 |
|  | PI-based/other | 7 | 1109 (529-2326) | 0.33 | 0.15-0.75 |  | 0.47 | | 0.19-1.13 |  |
| Immune suppression for age | Not severe | 6 | 1002 (450-2230) | 0.20 | 0.08-0.47 | <0.001 | 0.36 | 0.15-0.90 | | 0.006 |
|  | Severe | 36 | 5006 (3611-6940) | 1.00 | - |  | 1.00 | - | |  |
|  | Unknown | 1 | 250 (35-1774) | 0.05 | 0.01-0.37 |  | 0.09 | 0.01-0.62 | |  |
| Viral load (c/mL) | ≤100,000 | 12 | 2083 (1183-3667) | 0.63 | 0.31-1.27 | 0.260 | 0.93 | 0.44-1.99 | | 0.838 |
|  | >100,000 | 21 | 3349 (2183-5136) | 1.00 | - |  | 1.00 | - | |  |
|  | Unknown | 10 | 1942 (1045-3610) | 0.59 | 0.28-1.24 |  | 1.25 | 0.53-2.95 | |  |
| Weight-for-age z-score | >0 | 2 | 725 (181-2899) | 0.18 | 0.04-0.77 | <0.001 | 0.54 | | 0.11-2.61 | 0.084 |
|  | -3 to 0 | 25 | 3971 (2683-5877) | 1.00 | - |  | 1.00 | | - |  |
|  | <-3 | 7 | 13210 (6297-27708) | 3.30 | 1.42-7.66 |  | 2.58 | | 0.90-7.42 |  |
|  | Unknown | 9 | 1184 (616-2277) | 030 | 0.14-0.64 |  | 0.60 | | 0.23-1.58 |  |

Notes:

The following variables were excluded from the multivariable model due to correlation: year of birth (with age and also year of cART initiation); place of birth (with country group); ethnicity (with country group).
