# Supplementary material for: Long-term trends in mortality and AIDS-defining events after combination ART initiation among children and adolescents with perinatal HIV infection in 17 middle- and high-income countries in Europe and Thailand: A cohort study
Source: PLoS Med. 2018 Jan 30;15(1):e1002491. doi: 10.1371/journal.pmed.1002491 (PMC5790238; doi:10.1371/journal.pmed.1002491)
Supplement: S2 Table — (DOCX) [file pmed.1002491.s006.docx]

S2 Table: Rates and risk factors (baseline and time-updated) for death after 6 months of cART, using weight-for-age instead of BMI-for-age

|  | | **N deaths** | **Rate per 100,000 PY (95% CI)** | **Univariable** | | | **Multivariable** | | |
| --- | --- | --- | --- | --- | --- | --- | --- | --- | --- |
|  |  |  |  | **HR** | **95% CI** | **p** | **HR** | **95% CI** | **p** |
| **Baseline characteristics at initiation of cART** | | | | | | | | | |
| Sex | Male | 21 | 232 (151-356) | 0.74 | 0.42-1.29 | 0.290 | 0.84 | 0.47-1.52 | 0.569 |
|  | Female | 30 | 306 (214-437) | 1.00 | - |  | 1.00 | - |  |
| Age (per year increase) | <2 years | 9 | 155 (81-298) | 0.80 | 0.47-1.38 | <0.001 | 0.73 | 0.35-1.52 | 0.411 |
|  | ≥2 years | 42 | 322 (238-435) | 1.18 | 1.09-1.27 |  | 1.07 | 0.97-1.19 |  |
| Year of birth (per year increase) | <1995 | 20 | 567 (366-878) | 0.98 | 0.82-1.17 | <0.001 | - | | |
|  | ≥1995-<2000 | 19 | 270 (172-423) | 0.72 | 0.58-0.89 |  |  |  |  |
|  | ≥2000 | 12 | 145 (82-255) | 1.06 | 0.88-1.27 |  |  |  |  |
| Place of birth | Within country | 37 | 302 (219-417) | 1.00 | - | 0.066 | - | | |
|  | Abroad | 9 | 159 (83-305) | 0.50 | 0.24-1.04 |  |  |  |  |
|  | Unknown | 5 | 531 (221-1277) | 1.71 | 0.67-4.38 |  |  |  |  |
| Ethnicity | Black African | 35 | 7447 (5347-10372) | 0.56 | 0.35-0.90 | 0.012 | - | | |
|  | Asian | 35 | 13250 (9514-18455) | 1.00 | - |  |  |  |  |
|  | Other | 25 | 6573 (4441-9727) | 0.50 | 0.30-0.83 |  |  |  |  |
|  | Unknown | 15 | 5899 (3557-9786) | 0.45 | 0.24-0.82 |  |  |  |  |
| Country group | W&CE | 18 | 142 (90-226) | 0.25 | 0.13, 0.45 | <0.001 | 0.44 | 0.21-0.93 | 0.032 |
|  | EE&T | 33 | 531 (377-747) | 1.00 | - |  | 1.00 | - |  |
| AIDS diagnosis | No AIDS | 37 | 245 (178-338) | 1.00 | - | 0.218 | 1.00 | - | 0.493 |
|  | AIDS | 14 | 372 (221-629) | 1.48 | 0.79-2.75 |  | 1.25 | 0.66-2.36 |  |
| Year of cART initiation (per year increase) | | - | - | 0.62 | 0.39-0.99 | 0.044 | 0.65 | 0.37-1.12 | 0.121 |
| Initial regimen | NNRTI-based | 43 | 328 (243-442) | 1.00 | - | 0.018 | 1.00 | - | 0.548 |
|  | PI-based/other | 8 | 139 (70-279) | 0.40 | 0.19-0.86 |  | 0.77 | 0.32-1.82 |  |
| Immune suppression for age | Not severe | 5 | 85 (35-204) | 0.26 | 0.10-0.68 | 0.019 | 0.71 | 0.24-2.13 | 0.792 |
|  | Severe | 30 | 357 (250-511) | 1.00 | - |  | 1.00 | - |  |
|  | Unknown | 16 | 349 (214-570) | 1.00 | 0.54-1.84 |  | 0.79 | 0.33-1.91 |  |
| Viral load (c/mL) | ≤100,000 | 13 | 214 (124-369) | 0.94 | 0.45-1.96 | 0.247 | 0.71 | 0.33-1.51 | 0.670 |
|  | >100,000 | 17 | 241 (150-387) | 1.00 | - |  | 1.00 | - |  |
|  | Unknown | 21 | 366 (239-562) | 1.57 | 0.82-2.99 |  | 0.86 | 0.39-1.86 |  |
| Weight-for-age z-score | >0 | 2 | 68 (17-274) | 0.20 | 0.05-0.86 | 0.159 | 0.57 | 0.11-2.88 | 0.875 |
|  | -3 to 0 | 24 | 324 (217-483) | 1.00 | - |  | 1.00 | - |  |
|  | <-3 | 3 | 457 (147-1417) | 1.36 | 0.40-4.62 |  | 1.07 | 0.32-3.63 |  |
|  | Unknown | 22 | 280 (184-425) | 0.86 | 0.48-1.55 |  | 1.21 | 0.46-3.15 |  |
| **Time updated characteristics** | | | | | | | | | |
| Age (per year increase) | <5 years | 7 | 186 (89-390) | 0.65 | 0.45-0.95 | <0.001 | - | | |
|  | ≥5 years | 44 | 292 (217-292) | 1.22 | 1.13-1.32 |  |  |  |  |
| Immune suppression for age | Not severe | 9 | 67 (35-128) | 0.02 | 0.01-0.05 | <0.001 | 0.07 | 0.03-0.18 | <0.001 |
|  | Severe | 30 | 2617 (1830-3743) | 1.00 | - |  | 1.00 | - |  |
|  | Unknown | 12 | 285 (162-502) | 0.10 | 0.05-0.20 |  | 0.30 | 0.10-0.91 |  |
| Viral load (c/mL) | ≤400 | 12 | 102 (58-179) | 0.10 | 0.05-0.21 | <0.001 | 0.33 | 0.14-0.77 | 0.024 |
|  | >400 | 25 | 958 (647-1418) | 1.00 | - |  | 1.00 | - |  |
|  | Unknown | 14 | 315 (187-532) | 0.33 | 0.17-0.65 |  | 0.66 | 0.19-2.23 |  |
| % time since cART initiation with VL≤400c/mL | <80% | 30 | 477 (334-682) | 1.00 | - | <0.001 | - | | |
|  | ≥80% | 7 | 86 (41-181) | 0.18 | 0.08-0.41 |  |  |  |  |
|  | Unknown | 14 | 315 (187-532) | 0.67 | 0.35-1.28 |  |  |  |  |
| Weight-for-age z-score | >0 | 8 | 130 (65-260) | 0.40 | 0.16-0.99 | <0.001 | 0.75 | 0.26-2.16 | <0.001 |
|  | -3 to 0 | 14 | 230 (136-388) | 1.00 | - |  | 1.00 | - |  |
|  | <-3 | 12 | 9006 (5115-15859) | 41.33 | 19.82-86.17 |  | 17.25 | 6.60-45.09 |  |
|  | Unknown | 17 | 262 (163-421) | 0.56 | 0.26-1.19 |  | 0.57 | 0.20-1.63 |  |

Notes:

The following variables were excluded from the multivariable model due to correlation: year of birth (with age and also year of cART initiation); place of birth (with country group); ethnicity (with country group); time updated age (with age and also year of cART initiation); proportion of time with VL≤400c/mL (with time updated viral load),
