# Supplementary material for: Long-term trends in mortality and AIDS-defining events after combination ART initiation among children and adolescents with perinatal HIV infection in 17 middle- and high-income countries in Europe and Thailand: A cohort study
Source: PLoS Med. 2018 Jan 30;15(1):e1002491. doi: 10.1371/journal.pmed.1002491 (PMC5790238; doi:10.1371/journal.pmed.1002491)
Supplement: S3 Table — (DOCX) [file pmed.1002491.s007.docx]

S3 Table: Rates and baseline risk factors for first AIDS-defining event within 6 months after cART initiation

|  | | **N AIDS diagnoses** | **Rate per 100,000 PY (95% CI)** | **Univariable** | | | **Multivariable** | | | |
| --- | --- | --- | --- | --- | --- | --- | --- | --- | --- | --- |
|  |  |  |  | **HR** | **95% CI** | **p** | **HR** | **95% CI** | | **p** |
| **Baseline characteristics at initiation of cART** | | | | | | | | | | |
| Sex | Male | 51 | 7870 (5981-10355) | 0.96 | 0.66-1.40 | 0.842 | 0.93 | 0.64-1.36 | 0.721 | |
|  | Female | 59 | 8187 (6343-10566) | 1.00 | - |  | 1.00 | - |  |  |
| Age (per year increase) | <2 years | 37 | 7393 (5878-9300) | 0.83 | 0.61-1.13 | 0.123 | 0.71 | 0.50-1.00 | 0.075 | |
|  | ≥2 years | 73 | 9702 (7030-13391) | 0.98 | 0.93-1.03 |  | 1.00 | 0.95-1.07 |  |  |
| Year of birth (per year increase) | | - | - | 1.01 | 0.97-1.04 | 0.751 | - | | | |
| Place of birth | Within country | 72 | 8339 (6619-10506) | 1.00 | - | 0.881 | - | | | |
|  | Abroad | 33 | 7491 (5326-10537) | 0.90 | 0.60-1.36 |  |  |  |  |  |
|  | Unknown | 5 | 7717 (3212-18541) | 0.93 | 0.37-2.34 |  |  |  |  |  |
| Ethnicity | Black African | 35 | 7447 (5347-10372) | 0.56 | 0.35-0.90 | 0.012 | - | | | |
|  | Asian | 35 | 13250 (9514-18455) | 1.00 | - |  |  |  |  |  |
|  | Other | 25 | 6573 (4441-9727) | 0.50 | 0.30-0.83 |  |  |  |  |  |
|  | Unknown | 15 | 5899 (3557-9786) | 0.45 | 0.24-0.82 |  |  |  |  |  |
| Country group | W&CE | 53 | 6079 (4644-7957) | 0.53 | 0.37-0.78 | 0.001 | 0.68 | 0.44-1.05 | 0.079 | |
|  | EE&T | 57 | 11472 (8849-14873) | 1.00 | - |  | 1.00 | - |  |  |
| Year of cART initiation (per year increase) | | - | - | 0.87 | 0.69-1.11 | 0.262 | 0.89 | 0.70-1.14 | 0.358 | |
| Initial regimen | NNRTI-based | 75 | 8581 (6843-10761) | 1.00 | - | 0.334 | 1.00 | - | 0.327 | |
|  | PI-based/other | 35 | 7074 (5079-9853) | 0.82 | 0.55-1.23 |  | 0.81 | 0.53-1.24 |  |  |
| Immune suppression for age | Not severe | 26 | 4976 (3388-7309) | 0.50 | 0.31-0.80 | 0.009 | 0.55 | 0.34-0.91 | 0.046 | |
|  | Severe | 52 | 10030 (7643-13163) | 1.00 | - |  | 1.00 | - |  |  |
|  | Unknown | 32 | 9760 (6902-13801) | 0.98 | 0.63-1.52 |  | 0.66 | 0.38-1.14 |  |  |
| Viral load (c/mL) | ≤100,000 | 20 | 4064 (2622-6299) | 0.45 | 0.27-0.77 | <0.001 | 0.56 | 0.32-0.99 | 0.110 | |
|  | >100,000 | 42 | 9019 (6666-12204) | 1.00 | - |  | 1.00 | - |  |  |
|  | Unknown | 48 | 11681 (8803-15500) | 1.31 | 0.86-1.98 |  | 1.08 | 0.62-1.88 |  |  |
| BMI-for-age z-score | >0 | 13 | 3563 (2069-6136) | 0.51 | 0.26-1.00 | <0.001 | 0.66 | 0.33-1.30 | 0.011 | |
|  | -3 to 0 | 24 | 7025 (4709-10481) | 1.00 | - |  | 1.00 | - |  |  |
|  | <-3 | 6 | 22184 (9966-49378) | 3.11 | 1.26-7.69 |  | 2.49 | 1.05-6.40 |  |  |
|  | Unknown | 67 | 10548 (8302-13401) | 1.51 | 0.94-2.41 |  | 1.52 | 0.89-2.58 |  |  |

Notes:

The following variables were excluded from the multivariable model due to correlation: year of birth (with age and also year of cART initiation); place of birth (with country group); ethnicity (with country group).

10 children who died without an AIDS diagnosis are included.
