# Supplementary material for: Long-term trends in mortality and AIDS-defining events after combination ART initiation among children and adolescents with perinatal HIV infection in 17 middle- and high-income countries in Europe and Thailand: A cohort study
Source: PLoS Med. 2018 Jan 30;15(1):e1002491. doi: 10.1371/journal.pmed.1002491 (PMC5790238; doi:10.1371/journal.pmed.1002491)
Supplement: S4 Table — (DOCX) [file pmed.1002491.s008.docx]

S4 Table: Rates and risk factors (baseline and time-updated) for first AIDS-defining event after 6 months of cART

|  | | **N AIDS diagnoses** | **Rate per 100,000 PY (95% CI)** | **Univariable** | | | **Multivariable** | | |
| --- | --- | --- | --- | --- | --- | --- | --- | --- | --- |
|  |  |  |  | **HR** | **95% CI** | **p** | **HR** | **95% CI** | **p** |
| **Baseline characteristics at initiation of cART** | | | | | | | | | |
| Sex | Male | 74 | 1101 (876-1382) | 1.04 | 0.75, 1.43 | 0.831 | 1.02 | 0.74-1.39 | 0.977 |
|  | Female | 78 | 1083 (867, 1352) | 1.00 | - |  | 1.00 | - |  |
| Age (per year increase) | | - | - | 1.03 | 0.99-1.07 | 0.143 | 1.00 | 0.96-1.05 | 0.455 |
| Year of birth (per year increase) | <1999 | 79 | 1170 (939-1459) | 0.88 | 0.83, 0.94 | <0.001 | - | | |
|  | ≥1999-<2003 | 34 | 843 (602-1179) | 1.16 | 1.00-1.33 |  |  |  |  |
|  | ≥2003 | 39 | 1242 (907-1670) | 0.88 | 0.77-1.02 |  |  |  |  |
| Place of Birth | Within country | 111 | 1267 (1052, 1526) | 1.00 | - | 0.001 | - | | |
|  | Abroad | 29 | 654 (455, 941) | 0.50 | 0.33, 0.75 |  |  |  |  |
|  | Unknown | 12 | 1643 (933, 2893) | 1.35 | 0.74, 2.45 |  |  |  |  |
| Ethnicity | Black African | 32 | 643 (455, 909) | 0.23 | 0.15, 0.35 | <0.001 | - | | |
|  | Asian | 77 | 2563 (2050, 3204) | 1.00 | - |  |  |  |  |
|  | Other | 32 | 998 (706, 1411) | 0.33 | 0.22, 0.50 |  |  |  |  |
|  | Unknown | 11 | 402 (223, 726) | 0.15 | 0.08, 0.28 |  |  |  |  |
| Country group | W&CE | 51 | 532 (404. 700) | 0.24 | 0.17, 0.34 | <0.001 | *0.83 | 0.40-1.76 | 0.636 |
|  | EE&T | 101 | 2327 (1915, 2828) | 1.00 | - |  | 1.00 | - |  |
| Year of cART initiation (per year increase) | | - | - | 0.64 | 0.51-0.81 | <0.001 | 0.66 | 0.52-0.84 | 0.011 |
| Initial regimen | NNRTI-based | 124 | 1274 (1068-1519) | 1.00 | - | <0.001 | 1.00 | - | 0.028 |
|  | PI-based/other | 28 | 668 (461-968) | 0.46 | 0.31-0.70 |  | 0.63 | 0.40-1.00 |  |
| Immune suppression for age | Not severe | 26 | 532 (362-782) | 0.47 | 0.29-0.74 | <0.001 | 0.92 | 0.56-1.52 | 0.723 |
|  | Severe | 61 | 1089 (848-1400) | 1.00 | - |  | 1.00 | - |  |
|  | Unknown | 65 | 1889 (1481-2408) | 1.78 | 1.25-2.51 |  | 1.40 | 0.92-2.14 |  |
| Viral load (c/mL) | ≤100,000 | 20 | 403 (260-624) | 0.47 | 0.27-0.80 | <0.001 | 0.50 | 0.29-0.85 | <0.001 |
|  | >100,000 | 40 | 830 (609-1131) | 1.00 | - |  | 1.00 | - |  |
|  | Unknown | 92 | 2222 (1811-2726) | 2.65 | 1.83-3.85 |  | 1.39 | 0.85-2.27 |  |
| BMI-for-age z-score | >0 | 15 | 380 (229-631) | 0.40 | 0.22-0.74 | <0.001 | 0.50 | 0.26-0.93 | 0.091 |
|  | -3 to 0 | 34 | 943 (674-1320) | 1.00 | - |  | 1.00 | - |  |
|  | <-3 | 6 | 2136 (960-4754) | 2.26 | 0.94-5.45 |  | 1.02 | 0.39-2.70 |  |
|  | Unknown | 97 | 1592 (1304-1942) | 1.60 | 1.09-2.37 |  | 0.70 | 0.42-1.16 |  |
| **Time updated characteristics** | | | | | | | | | |
| Age (per year increase) | <10 years | 64 | 970 (760-1240) | 0.98 | 0.02-1.04 | 0.512 | - | | |
|  | ≥10 years | 88 | 1200 (974-1479) | 1.10 | 1.01-1.19 |  |  |  |  |
| Immune suppression for age | Not severe | 63 | 636 (497-814) | 0.17 | 0.11-0.26 | <0.001 | - | | |
|  | Severe | 36 | 4672 (3370-6477) | 1.00 | - |  |  |  |  |
|  | Unknown | 53 | 1632 (1247-2137) | 0.39 | 0.25-0.61 |  |  |  |  |
| Effect of time updated immune suppression for age among those in W&CE: | | | | | | Not severe | 0.84 | 0.51-1.38 | Test for interaction:  p=0.015 |
|  |  |  |  |  |  | Severe | 1.00 | - |  |
|  |  |  |  |  |  | Unknown | 1.02 | 0.67-1.56 |  |
| Effect of time updated immune suppression for age among those in EE&T: | | | | | | Not severe | 0.38 | 0.21-0.68 |  |
|  |  |  |  |  |  | Severe | 1.00 | - |  |
|  |  |  |  |  |  | Unknown | 0.42 | 0.21-0.84 |  |
| Viral load (c/mL) | ≤400 | 50 | 576 (437-760) | 0.34 | 0.22-0.53 | <0.001 | 0.53 | 0.33-0.85 | 0.002 |
|  | >400 | 33 | 1730 (1230-2434) | 1.00 | - |  | 1.00 | - |  |
|  | Unknown | 69 | 2064 (1630-2613) | 1.11 | 0.73-1.69 |  | 0.92 | 0.50-1.68 |  |
| % time since cART initiation with VL≤400c/mL | <80% | 65 | 1435 (1125-1830) | 1.00 | - | <0.001 | - | | |
|  | ≥80% | 18 | 297 (187-472) | 0.22 | 0.13-0.37 |  |  |  |  |
|  | Unknown | 69 | 2064 (1630-2613) | 1.37 | 0.97-1.92 |  |  |  |  |
| BMI-for-age z-score | >0 | 25 | 549 (371-813) | 0.69 | 0.41-1.18 | <0.001 | 1.36 | 0.78-2.40 | <0.001 |
|  | -3 to 0 | 31 | 714 (502-1015) | 1.00 | - |  | 1.00 | - |  |
|  | <-3 | 11 | 16983 (9405-30666) | 26.90 | 13.39-54.07 |  | 14.44 | 6.32-32.97 |  |
|  | Unknown | 85 | 1711 (1384-2117) | 1.99 | 1.31-3.00 |  | 2.42 | 1.42-4.14 |  |

Notes:

The following variables were excluded from the multivariable model due to correlation: year of birth (with age and also year of cART initiation); place of birth (with country group); ethnicity (with country group); time updated age (with age and also year of cART initiation); proportion of time with VL≤400c/mL (with time updated viral load),

15 children who died without an AIDS diagnosis are included

*Represents the effect of country group among those with severe time-updated immune suppression for age.
